# Evaluating the adequacy of current dietary guidelines for seafood as a source of long-chain omega-3 fatty acids

**DOI:** 10.1038/s41598-026-41320-w

**Published:** 2026-03-20

**Authors:** M. Sprague, M. B. Betancor, A. Rolland, D. R. Tocher

**Affiliations:** 1 Faculty of Natural Sciences, Institute of Aquaculture, University of Stirling, Stirling, FK9 4LA Scotland, UK; 2 Guangdong Provincial Key Laboratory of Marine Biotechnology, Institute of Marine Sciences, Shantou University, Shantou, 515063 China

**Keywords:** Public health, Fish consumption, Aquaculture, EPA and DHA, Policy making, Nutrition, Fatty acids, Public health

## Abstract

Seafood is widely encouraged as part of a healthy diet due to its rich source of key nutrients, with guidelines advocating two portions per week, one of which is oily. As the main dietary provider of the health-promoting omega-3 long-chain polyunsaturated fatty acids, eicosapentaenoic (EPA) and docosahexaenoic (DHA) acids, seafood consumption is essential to achieve recommended EPA + DHA intake levels, which range from 0.25 > 1.0 g.day^− 1^, to support optimal health. Here we examine the lipid and fatty acid contents of seafood in relation to EPA + DHA recommendations. With more seafood being farmed we found that, despite the industrialisation process resulting in an increased *n*-6/*n*-3 ratio, farmed fish still provide similar or greater amounts of EPA + DHA than their wild equivalents. However, only a single 140 g portion of Atlantic mackerel would meet the UK’s recommended 3.15 g EPA + DHA intake. Of the available combinations analysed, 75% of any two portions would not deliver the lower European Safety Authority’s (EFSA) recommendation of 1.75 g.EPA + DHA.week^− 1^, whereas following current advice of two portions per week, where at least one is oily, resulted in 75% of combinations satisfying the lower EFSA amount but just 11% fulfilling the UK’s higher recommendation. While actual seafood intake remains below recommended amounts, necessitating further consumer education, changing the current guidelines to encourage the consumption of three portions of seafood per week, whereby two are oily, may prove challenging but would ensure that 99% and 75% of combinations surpass EFSA and UK recommendations, respectively, potentiating improved public health outcomes.

**Supplementary Information:**

The online version contains supplementary material available at 10.1038/s41598-026-41320-w.

## Introduction

Throughout history seafood consumption has been associated with having a beneficial effect on health. The ancient Romans and Greeks, for example, would prepare fermented fish sauce, garum, which was often dispensed to treat all manner of ailments. The Vikings valued fish and fish livers for bestowing vitality. In the mid-1700’s English physicians Samuel Key, and then Thomas Percival, began administering cod liver oil to treat chronic rheumatoid arthritis^1^. Widespread use of cod liver oil then followed for remedying various conditions including rheumatism and rickets, effectively establishing the modern-day fish oil industry. However, while improved health outcomes were realised, the factors causing them remained unclear. Research in the early twentieth century started to shed light on the importance of some of the nutritional properties of food with the discovery of nutrients such as vitamins and the role of fat in the diet. Studies by George and Mildred Burr demonstrated the requirement for two specific fatty acids, the omega-6 (*n*-6) linoleic (LA, 18:2*n*-6) and the omega-3 (*n*-3) α-linolenic (ALA, 18:3*n*-3) acids^2,3^. These essential fatty acids (EFA) are unable to be synthesised de novo by vertebrates, including humans, owing to the absence of the necessary desaturases required to convert oleic acid (OA, 18:1*n*-9) to LA and/or ALA, and must therefore be included within the diet.

As the incidence of non-communicable diseases (NCDs) such as cardiovascular, inflammatory disorders and cancer among others increased, especially within westernised countries, researchers began to establish a link between diet and human health. Hugh Sinclair in the UK and Ancel Keys in America in particular, began to question the role of dietary fat and EFA deficiency in human nutrition^4,5^. Sinclair^6^ studied the diets of Canadian Indians and the Inuit population noting the nutritional values of their indigenous aliments, particularly the Inuit high fat marine diet, and the impact of European diets on their health. However, it was the seminal study by Bang et al.^7^ that transformed the field of human nutrition with their observations of low incidence of ischaemic heart disease in Greenland Innuit being related to diet, specifically their high intake of long-chain polyunsaturated fatty acids (LC-PUFA) from a marine-based diet. This, and follow-up studies, suggested that the presence of the *n*-3 LC-PUFA, eicosapentaenoic (EPA, 20:5*n*-3) and docosahexaenoic (DHA, 22:6*n*-3) acids, found almost exclusively in marine foods, were primarily responsible for the range of health benefits of a seafood diet through their molecular, cellular and physiological actions as well as their importance in the development and function of neural tissues^8–10^. Although humans can synthesise both EPA and DHA from the shorter chain *n*-3 metabolic precursor, ALA, conversion is nonetheless quite low, even after considering individual variables such as diet, sex, age, etc.^11–13^. While endogenous production may meet critical requirements^14^, dietary intake of preformed EPA and DHA is encouraged to provide optimal requirements in preventing NCDs^9,12,15^.

In an intervention study by Burr et al.^16^, individuals recovering from myocardial infarction who increased oily fish intake significantly decreased their risk of reinfarction or death than control subjects. This supported findings from epidemiological studies including a 20-year Dutch study showing reduced mortality from coronary heart disease in men with higher fish consumption^17^. Studies such as these were influential in the UK’s Department of Health’s Committee on Medical Aspects of Food Policy issuing the 1994 recommendation that the population should consume at least two portions of fish per week, of which at least one should be oily^18^, a recommendation that has since been adopted globally^19–22^. While guidelines may stipulate fish consumption this does not necessarily exclude other seafood such as shellfish, although the tendency to acquire marine biotoxins more readily may explain the reason for their omission. Nevertheless, while there is general agreement for regular fish consumption there can be distinct differences in suggested portion sizes e.g. Europe 20–200 g, UK 140 g, USA 113 g^19–21,23^. Similarly, there is no global consensus on a recommended EPA and DHA intake (Table 1), which may be due to this varying depending upon the health outcome assessed^24^. Notwithstanding, there is still disagreement when based on one outcome, such as the prevention of cardiovascular disease. For example, the European Food Safety Authority (EFSA) advises 0.25 g EPA + DHA.day^−1^^25^, whereas the American Heart Association has no recommendation for the ordinary public, other than to follow fish consumption guidelines, but recommends that existing sufferers of coronary heart disease consume 1 g.day^−1^^26^. The Global Organization for EPA and DHA omega-3’s (GOED), an industry representative for the *n*-3 market, strongly advocates the 0.5 g.day^−1^ intake recommended by the International Society for the Study of Fatty Acids and Lipids (ISSFAL) for optimal cardiac health in adults^24,27,28^. However, just 20% of the global population is estimated to consume > 0.25 g EPA + DHA daily^29^. Both the UK and USA experience higher mortalities (~ 90%) from NCDs than the global average (74%), with 25–30% attributable to cardiovascular disease^30^. In Europe alone, almost a quarter of a million deaths recorded in 2016 from cardiovascular disease were credited to a diet low in seafood *n*-3^31^. Regular seafood consumption can therefore contribute to mitigating against these risks while also reducing the burden on national health services^32,33^.

**Table 1:** Examples of global recommendations on fish consumption, portion sizes and/or EPA + DHA (g.week^−1^) intake levels.

	Portion size (g)	Frequency (portions per week)	EPA + DHA day/week
*International*			
ISFAL^27^	–	–	0.5 g.day^−1^ / 3.5 g.week^−1^
GOED^24^	–	–	0.5 g.day^−1^ / 3.5 g.week^−1^
WHO^22^	–	2 portions, at least 1 oily	0.25.day^−1^ / 1.75 g.week^−1^
*Europe*			
EFSA^19,25^	130	2 portions, at least 1 oily	0.25 g.day^−1^ / 1.75 g.week^−1^
Individual states see^20^	20–200	1–7 portions to include oily fish	–
*United Kingdom*			
SACN/COT^18,23^	140	2 portions, at least 1 oily	0.45 g.day^−1^ / 3.15 g.week^−1^
*United States*			
AHA^26^	85	2 portions, particularly fatty fish	None* 1 g.day^−1^ / 7 g.week^-1#^
USDA^21^	113	2 portions, at least 1 oily	–

– no formal recommendation provided.

*No recommendation for the ordinary public other than following fish consumption guidelines.

^#^For those with a history of coronary heart disease.

In 2004, the UK’s Scientific Advisory Committee on Nutrition (SACN) and Committee on Toxicity (COT) further endorsed their earlier recommendation on fish consumption, adding that this should provide ~ 0.45 g EPA + DHA.day^−1^^23^. However, to date, the prospect of attaining this target through seafood consumption alone has not been examined. With a widening choice of seafood, climate change predicted to affect marine LC-PUFA production^34^, and changes to culture practices influencing the nutritional composition of farmed foods^35–38^, the continual assessment of our available seafood is important to ensure that recommendations satisfy our nutrient requirements. The present study therefore examined the lipid and fatty acid compositions, particularly with respect to EPA and DHA contents, of the different types of seafood available to consumers, and places into context the required intake to attain recommended levels which policy makers, health nutritionists and ultimately consumers should find of interest.

## Results and discussion

### Lipid content

**Fig. 1:**
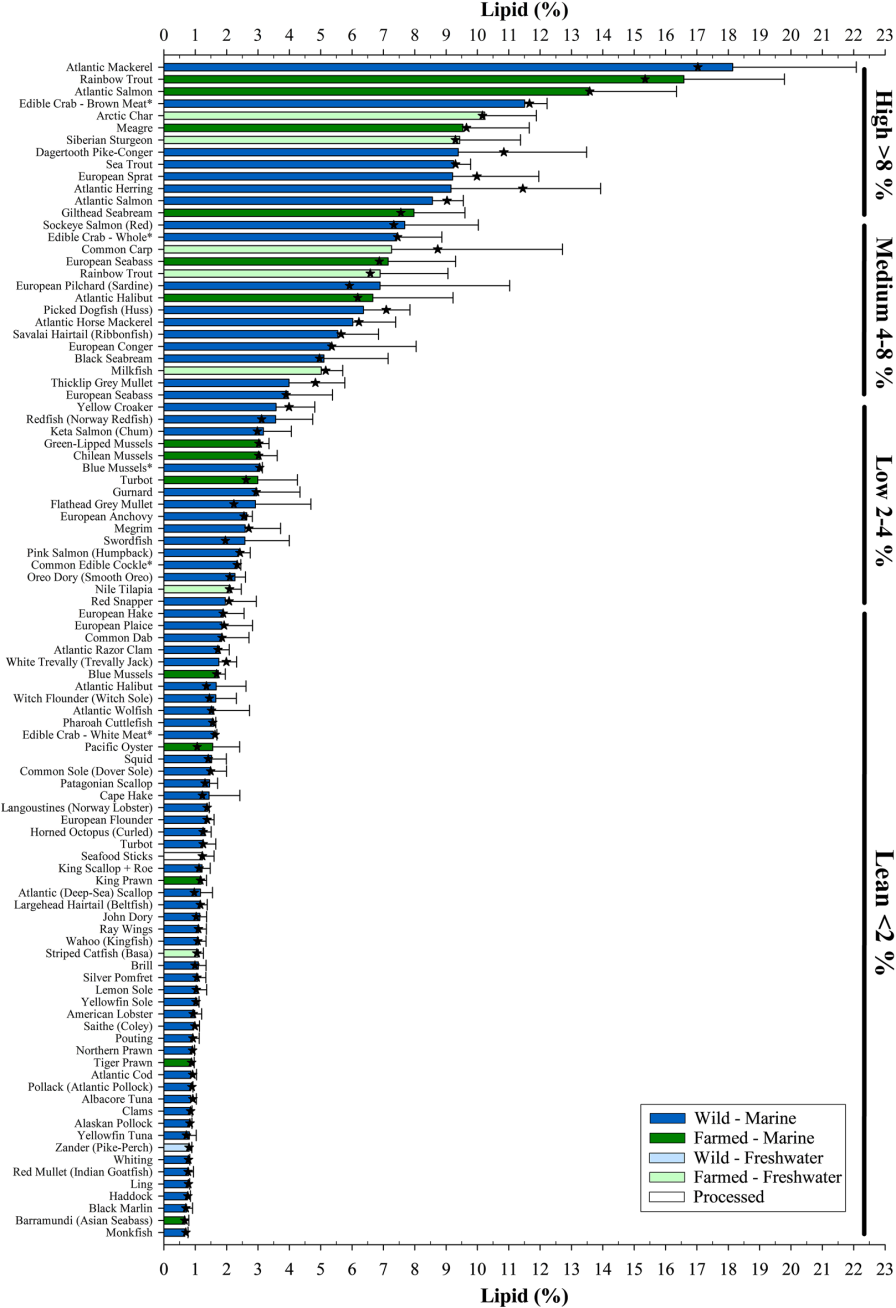
Lipid content (%, ww) of wild and/or farmed seafood of marine and/or freshwater origin. Samples ranked in descending order. ★ indicates median lipid value. Bold lines at top of data bars denote Lean (< 2%), Low (2–4%), Medium (4–8%) and High Fat (> 8%) lipid categories, based on Ackman^39^. All samples analysed were raw unless denoted by *. Refer to Supplementary Table 1 for further sample information.

Seafood is typically classed as oily or non-oily based on their flesh lipid (fat) content. Ackman^39^ arbitrarily used four distinct categories consisting of Lean (< 2%), Low (2–4%), Medium (4–8%) and High Fat (> 8%) to separate different fish species. Applying the same criteria to the current dataset resulted in most seafood samples (52 of 97) being ranked as lean, with a further 17, 15 and 13 assigned as low, medium and high fat, respectively, with levels ranging from 0.7 ± 0.1% (mean ± SD) in monkfish (*Lophius piscatorius*) to 18.1 ± 3.9% in Atlantic mackerel (*Scomber scombrus*) (Fig. 1). Shellfish and molluscs were generally ranked within the lean (< 2%) and low-fat (2–4%) categories. The only non-fish species featuring outwith this range was the edible crab (*Cancer pagurus*) owing to brown meat from the main body, which contains both digestive glands (hepatopancreas) and reproductive organs, having a higher lipid content (11.5 ± 0.7%) as compared to white meat (1.6 ± 0.1%) found in claws and legs. Predictably, given that two-thirds of the meat yield from edible crab is brown^40^, whole crab (mixture of brown and white meats) gave an intermediary lipid value (7.4 ± 1.5%). Lipids, specifically the fatty acids they comprise, are the major source of metabolic energy for both growth and reproduction in fish^41^. Excess lipid is preferentially stored in depot organs including muscle, liver and mesenteric tissue, and varies in content and storage organ according to species^42^. Among the high fat species, the salmonids Atlantic salmon (*Salmo salar*), rainbow trout (*Oncorhynchus mykiss*), sea trout (*Salmo trutta*) and Arctic char (*Salvelinus alpinus*) were prevalent. These species along with the likes of Atlantic mackerel and herring (*Clupea harengus*) store their lipid in the muscle, whereas the lean Atlantic cod (*Gadus morhua*), whose flesh lipid content was 0.9 ± 0.2%, preferentially stores excess lipid in the liver. Although cod liver and other tissues such as roe are eaten, the focus of the present study was on flesh, the more commonly consumed tissue.

It is important to note, however, that the lipid content of the flesh of fish can vary within and between individuals of the same species. Factors such as genetics, diet, age and/or body size, reproductive status, and season are all known to impact upon lipid storage^43–46^. Wild fish are therefore more susceptible to such changes in their flesh lipid profiles, and species normally considered as oily can be found to contain low levels. For example, Atlantic herring lipid contents ranged from a high of 14.4 to a low of 1.3% (mean 9.2 ± 4.8%, median 11.5%), reflecting their year-round availability and seasonality of lipid and nutritional profiles. From the consumer perspective, unless purchased fresh or at source, it may not always be easy to determine the season when fish were caught. For farmed fish this variation in lipid content is expected to be lower since they are generally reared to a uniform specification. Nevertheless, factors such as body size can still influence lipid status as evinced in rainbow trout reared in either freshwater (6.9 ± 2.2%) or marine (16.6 ± 3.2%) environments where typical harvest sizes are in the region of 400 g and 4 kg, respectively. On the other hand, in farmed common carp (*Cyprinus carpio*), variation in the fillet lipid content (mean 7.3 ± 5.5%, median 8.7%, range 1.8–14.8%) is more likely a result of a combination of factors including body size and dietary fat content related to issues surrounding both seasonality and sexual maturation and/or textural and organoleptic properties within carp farming^47,48^.

In addition to the above, the body location from where the flesh is sampled, particularly with respect to larger-sized fish, can also affect lipid levels. Tuna is often considered a fatty fish by consumers and dietary advisory bodies. However, both albacore (*Thunnus alalunga*) and yellowfin tuna (*T. albacores*) sampled in the present study contained lipid contents of 0.9 ± 0.2 and 0.8 ± 0.2%, respectively, towards the lower range of the 0.7–18.7% previously reported in albacore tuna^49^. This is primarily down to the type of cut readily made available to UK consumers, being the leaner part of the loin whereas the fattier sections such as belly (Toro) are often reserved and exported to the more lucrative sushi and sashimi markets where they are highly prized. Lipid contents are known to vary throughout the fillet, particularly in oily species, changing from rostral (head) to caudal (tail) and from dorsal (back) to ventral (belly) regions, but also in relation to the presence and proportions of red and white muscle in the cut^43,50–52^. Thus, as consumers rarely purchase a whole-side or entire large-sized fish, instead purchasing ready-preprepared cuts where it can be difficult to identify where on the fish they originated, the actual amount of lipid within a portion could vary considerably. From a human health perspective, limiting high-fat foods in the diet is typically recommended^18,21,25^. However, it is the composition of the lipid in terms of fatty acid content that influences the ‘healthiness’ of foods and why the inclusion of seafood in the diet with its unique source of *n*-3 LC-PUFA, EPA and DHA, is considered beneficial to human health.

### Fatty acid profiles

**Fig. 2:**
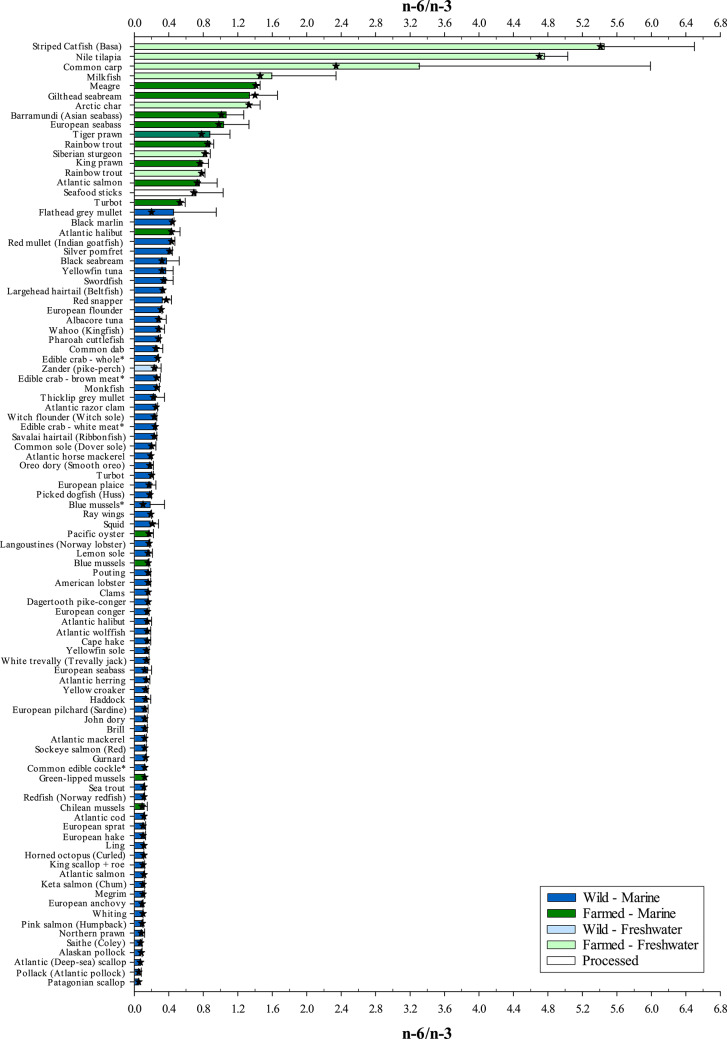
The *n*-6/*n*-3 ratio of wild and/or farmed seafood of marine and/or freshwater origin. Samples ranked in descending order. ★ indicates median lipid value. All samples analysed were raw unless denoted by *. Refer to Supplementary Table 1 for further sample information.

The association of changed dietary habits and increased non-communicable diseases was first recognised as far back as the mid-twentieth century^4,5^. In particular, increased intake of *n*-6 fatty acids coupled with decreased *n*-3 fatty acid intake, a result of the industrialisation of food production, led to the *n*-6/*n*-3 ratio being used as an index of health status, with poorer health linked to an imbalance of the ratio in favour of *n*-6^53^. In the current study, farmed fish and prawns presented higher *n*-6/*n*-3 ratios than wild fish (Fig. 2). While these species are fed formulated diets, other farmed seafood such as mussels and oysters are considered non-fed, obtaining their nutrients from the water where they are cultured and, therefore, presenting lower *n*-6/*n*-3 ratios. As with human nutrition, the industrialisation of farmed fish production has resulted in changes to ingredients used in feed formulations^35–38^. Vegetable oils including rapeseed, linseed (flaxseed), soyabean and camelina are utilised currently throughout the farmed seafood (aquaculture) sector with their inclusion rising year-on-year to satisfy the growing industry^35^. Given that flesh fatty acid profiles of fish generally reflect that of the dietary oil source^41^, i.e. you are what you eat, it is perhaps of little surprise that the increasing presence of plant-based oils in aquafeeds has resulted in a shift towards a higher *n*-6/*n*-3 ratio. A simple comparison of the fatty acid profiles of plant oils and animal fat sources of terrestrial origin reveal a higher level of *n*-6 than their marine counterparts owing to the generally high presence of LA (Table 2). Unlike vertebrates, higher plants are capable of desaturating OA to LA and ALA, with seed oils typically attaining a high proportion of LA^54^. As a result, the inclusion of oilseeds within animal feeds, and the human diet in general, have contributed to a high *n*-6/*n*-3 ratio whereby the high *n*-6 intake can potentially impede the metabolism of ALA^11^. While few plant oils present ratios in favour of *n*-3 fatty acids, camelina and linseed show *n*-6/*n*-3 ratios of 0.55 and 0.23, respectively (*n*-3/*n*-6 1.82 and 4.40, respectively), indicating that inclusion of these oils within feeds can reduce dietary *n*-6/*n*-3 ratios. Nevertheless, an expert panel convened by the UK’s Food Standards Agency concluded that, in terms of cardiovascular health, the dietary *n*-6/*n*-3 ratio is not a useful indicator^55^. The western diet is already awash with an abundance of *n*-6 fatty acids with the *n*-6/*n*-3 ratio suggested to be in the region of 15–20:1^53^. Moreover, the ratio fails to differentiate between the different types of fatty acids present^56^. Historically, *n*-6 fatty acids have been perceived as being pro-inflammatory, and therefore deemed ‘bad’, whereas the converse was said of *n*-3 fatty acids. However, the physiological functions of fatty acids differ individually such that some *n*-6 fatty acids are considered beneficial^57^. The *n*-6 LC-PUFA, arachidonic acid (ARA; 20:4*n*-6) and the *n*-3 EPA and DHA are important integral components of the cell membrane that control a cascade of lipid mediators involved in regulating cell responses. Thus, while traditional practices have been to group classes of fatty acids, an alternative approach would be to consider them on an individual basis.

**Table 2:** Fatty acid profiles (% of total fatty acids) of some common oils and fats from marine and terrestrial settings of animal or plant/protist origin.

	Marine											
	Animal									Protists		
	Capelin	Krill	Mackerel	Menhaden	Sardine	Seal	Tuna	Typical Northern hemisphere fish oil	Typical Southern hemisphere fish oil	*Nannochloropsis*	*Schizochytrium* (EPA + DHA strain)	*Schizochytrium* (DHA strain)
***Fatty acid***												
10:0	–	–	–	–	–	–	–	–	–	–	–	–
12:0	0.1	0.1	0.1	0.1	–	–	–	–	0.1	–	0.1	0.1
14:0	5.7	8.5	3.8	9.6	6.7	3.9	3.0	5.1	7.1	2.6	1.8	4.7
16:0	11.2	21.6	17.3	20.7	19.2	6.9	17.3	15.2	18.2	19.2	21.5	17.3
18:0	1.2	1.7	3.9	3.4	3.2	1.0	5.1	2.0	3.6	0.7	1.1	0.7
20:0	0.1	0.2	0.4	0.4	0.4	0.1	0.4	0.2	0.2	0.1	0.5	0.1
22:0	–	–	0.1	0.2	0.1	0.1	0.2	0.1	0.1	–	0.1	0.2
24:0	–	–	–	–	–	0.1	–	–	–	–	–	0.1
***Total saturates***^***1***^	***19.4***	***33.2***	***27.6***	***36.5***	***30.8***	***13.3***	***27.8***	***24.4***	***31.3***	***24.6***	***25.6***	***23.6***
16:1n-7	7.0	8.4	5.0	12.1	11.3	12.2	5.7	6.3	8.6	21.5	0.1	0.2
18:1n-9 (OA)	9.9	11.1	15.1	5.7	7.5	21.7	13.6	15.7	7.9	6.8	0.2	7.0
18:1n-7	2.6	5.9	3.1	3.0	3.9	4.4	2.8	2.4	3.0	0.6	–	0.2
20:1n-11	0.8	–	0.6	–	0.1	1.8	0.4	0.8	0.2	–	–	–
20:1n-9	15.6	1.1	4.4	0.8	1.3	12.0	2.1	6.9	1.4	–	–	–
22:1n-11	18.9	0.4	6.6	–	0.6	4.7	1.4	10.1	1.1	–	–	–
22:1n-9	2.4	0.7	0.8	0.1	0.2	0.8	0.2	0.8	0.2	–	–	–
24:1n-9	0.7	–	1.2	0.4	0.5	0.2	0.5	1.2	0.5	–	–	–
***Total monoenes***^***2***^	***59.0***	***28.3***	***38.0***	***22.5***	***25.8***	***60.0***	***28.0***	***45.3***	***24.0***	***34.6***	***0.3***	***7.5***
18:2n-6 (LA)	1.3	1.3	1.4	1.5	1.1	2.3	1.5	2.2	1.2	2.3	0.1	0.7
18:3n-6	0.1	0.2	–	0.4	0.4	0.1	0.1	0.1	0.4	0.5	–	0.2
20:2n-6?	0.2	–	0.4	0.2	0.1	0.3	0.2	0.4	0.2	0.1	–	–
20:4n-6 (ARA)	0.2	0.2	0.9	1.4	0.9	0.2	2.0	0.6	1.2	4.7	3.2	1.3
22:5n-6	–	–	0.5	0.6	0.2	0.1	1.8	0.2	0.5	–	2.9	17.1
***Total n-6 PUFA***^***3***^	***2.0***	***1.8***	***3.4***	***4.7***	***2.9***	***3.2***	***6.0***	***3.5***	***3.6***	***8.0***	***6.9***	***19.9***
18:3n-3 (ALA)	*0.7*	*0.7*	*1.0*	*1.6*	*0.7*	*0.9*	*0.7*	*1.6*	*0.9*	*0.1*	*0.1*	*0.1*
18:4n-3	*3.3*	*2.6*	*2.4*	*2.5*	*3.8*	*2.6*	*1.2*	*2.9*	*3.0*	*–*	*0.3*	*0.4*
20:3n-3	*0.1*	*–*	*0.2*	*0.2*	*0.1*	*0.1*	*0.1*	*0.1*	*0.1*	*–*	*0.2*	*0.1*
20:4n-3	*0.5*	*0.5*	*1.0*	*1.3*	*1.1*	*0.6*	*0.5*	*0.6*	*0.7*	*0.1*	*1.0*	*0.8*
20:5n-3 (EPA)	*7.2*	*21.2*	*8.4*	*12.5*	*19.2*	*5.7*	*7.3*	*8.3*	*18.0*	*32.2*	*19.6*	*1.6*
22:5n-3 (DPA)	*0.6*	*0.4*	*1.7*	*2.7*	*1.8*	*4.0*	*1.5*	*0.9*	*2.0*	*–*	*4.0*	*0.7*
22:6 n-3 (DHA)	*5.5*	*7.8*	*14.9*	*10.4*	*6.6*	*7.9*	*26.1*	*10.2*	*9.7*	*–*	*41.8*	*45.0*
***Total n-3 PUFA***^***4***^	***18.2***	***34.0***	***30.1***	***31.9***	***34.1***	***22.4***	***37.6***	***25.0***	***35.3***	***32.4***	***67.2***	***48.7***
***Total PUFA***^***5***^	***21.5***	***38.5***	***34.4***	***41.0***	***43.4***	***26.7***	***44.2***	***30.3***	***44.7***	***40.8***	***74.1***	***68.9***
***n-3/n-6***	***9.18***	***19.13***	***9.00***	***6.83***	***11.64***	***6.96***	***6.25***	***7.14***	***9.81***	***4.05***	***9.74***	***2.45***

	Terrestrial																						
	Animal					Plants																	
	Beef	Black soldier fly	Mealworm	Pork	Poultry (goose)	Almond	Avocado	Borage	Camelina	Coconut	Corn	Echium	Grapeseed	Groundnut	Linseed	Olive	Palm	Rapeseed	Rice bran	Sesame	Soya	Sunflower	Walnut
***Fatty acid***																							
10:0	0.1	0.1	–	–	–	–	–	–	–	1.1	–	–	–	–	–	–	–	–	–	–	–	–	–
12:0	–	33.0	–	0.1	0.2	–	–	0.1	–	45.8	–	–	–	–	0.1	0.1	–	0.1	–	0.1	–	–	0.1
14:0	3.0	8.7	2.3	1.2	0.5	0.1	0.1	0.1	0.1	24.5	–	0.1	0.1	0.1	0.1	–	0.5	0.1	0.3	0.1	0.1	0.1	0.1
16:0	26.1	17.6	17.2	26.4	20.2	5.5	13.7	10.7	5.5	13.1	11.8	5.8	6.9	11.0	5.0	10.5	36.7	4.5	19.0	9.9	10.4	7.1	6.9
18:0	23.3	2.9	2.5	16.7	5.0	2.0	2.7	3.7	2.8	4.3	1.8	2.9	4.0	3.5	4.3	3.1	7.6	1.6	2.1	4.7	4.5	3.1	2.6
20:0	0.2	0.1	0.1	0.2	–	0.1	0.5	0.2	1.2	0.1	0.5	0.1	0.1	1.6	0.1	0.6	0.5	0.6	0.9	0.6	0.5	0.2	0.1
22:0	–	0.1	–	–	–	0.1	0.1	0.1	0.3	–	0.1	–	–	3.1	0.1	0.1	–	0.3	0.3	0.1	0.5	0.6	–
24:0	–	–	–	–	–	0.1	–	0.1	0.1	–	0.2	–	0.1	1.8	0.2	0.1	–	0.1	0.5	0.2	0.2	0.2	–
***Total saturates***^***1***^	***55.5***	***62.6***	***22.2***	***44.8***	***25.8***	***7.9***	***17.1***	***15.1***	***10.1***	***88.8***	***14.4***	***9.0***	***11.3***	***21.1***	***10.0***	***14.5***	***45.2***	***7.3***	***23.2***	***15.7***	***16.1***	***11.4***	***9.8***
16:1n-7	2.5	3.4	1.7	2.3	3.0	0.6	2.5	0.2	0.1	–	0.1	0.1	0.1	0.1	0.1	0.6	.1	–	0.2	0.1	0.1	0.2	.1
18:1n-9 (OA)	34.3	14.0	40.9	37.7	54.0	65.6	64.7	16.1	14.3	8.9	29.5	14.1	18.4	54.9	19.1	69.7	41.7	58.7	41.2	38.5	20.2	26.0	15.7
18:1n-7	3.6	0.7	0.3	2.7	1.8	1.8	3.0	0.7	0.8	0.1	0.7	0.6	0.9	0.7	0.7	1.7	0.6	3.2	0.9	0.9	1.2	1.0	0.9
20:1n-11	0.2	–	–	–	0.1	0.1	–	–	–	–	–	–	–	–	–	–	–	–	–	–	–	–	–
20:1n-9	0.2	–	0.1	0.8	0.5	0.3	0.3	4.0	14.5	–	0.3	1.1	0.2	1.2	0.2	0.5	0.2	1.5	0.7	0.3	0.2	0.2	0.3
22:1n-11	–	–	–	–	–	–	–	–	–	–	–	0.1	–	0.1	–	–	–	–	–	–	–	–	–
22:1n-9	–	–	–	–	–	–	–	2.6	2.5	–	–	0.3	–	0.1	–	–	–	0.6	–	–	–	–	–
24:1n-9	–	–	–	–	–	–	–	1.7	0.6	–	–	0.2	–	–	–	0.2	–	0.2	–	–	–	–	–
***Total monoenes***^***2***^	***42.1***	***18.5***	***43.9***	***44.0***	***59.7***	***68.6***	***70.7***	***25.4***	***33.2***	***9.0***	***30.6***	***16.6***	***19.6***	***57.1***	***20.1***	***72.9***	***42.6***	***64.3***	***43.0***	***39.9***	***21.8***	***27.4***	***17.0***
18:2n-6 (LA)	1.4	16.3	31.8	9.0	13.4	22.5	11.4	37.4	18.0	2.2	54.1	26.3	68.7	21.7	12.9	11.7	12.0	19.1	32.6	43.9	54.3	61.1	59.8
18:3n-6	–	–	–	–	–	0.8	–	21.6	–	–	–	10.4	–	–	–	–	–	–	–	0.1	–	–	0.1
20:2n-6	–	–	0.1	0.5	0.1	–	–	0.2	2.0	–	–	0.1	–	–	–	–	–	1.1	–	–	–	–	–
20:4n-6 (ARA)	–	–	–	0.2	0.1	–	–	–	–	–	–	–	–	–	–	–	–	–	–	–	–	–	–
22:5n-6	–	–	–	–	–	–	–	–	–	–	–	–	–	–	–	–	–	–	–	–	–	–	–
***Total n–6 PUFA***^***3***^	***1.5***	***16.3***	***32.0***	***9.8***	***13.6***	***23.3***	***11.4***	***59.2***	***20.1***	***2.2***	***54.1***	***36.8***	***68.7***	***21.7***	***12.9***	***11.7***	***12.0***	***19.3***	***32.6***	***44.0***	***54.3***	***61.1***	***60.0***
18:3n-3 (ALA)	*0.5*	*–*	*1.7*	*0.8*	*0.9*	*–*	*0.8*	*0.2*	*35.1*	*–*	*0.9*	*29.0*	*0.5*	*0.1*	*57.1*	*0.8*	*0.2*	*9.1*	*1.2*	*0.5*	*7.7*	*0.1*	*12.5*
18:4n-3	*0.4*	*–*	*–*	*0.1*	*–*	*0.1*	*–*	*0.1*	*–*	*–*	*–*	*8.6*	*–*	*–*	*–*	*–*	*–*	*–*	*–*	*–*	*–*	*–*	*0.74*
20:3n-3	*–*	*–*	*–*	*0.1*	*–*	*0.1*	*–*	*–*	*1.5*	*–*	*–*	*–*	*–*	*–*	*–*	*–*	*–*	*–*	*–*	*–*	*–*	*–*	*–*
20:4n-3	*–*	*–*	*–*	*–*	*–*	*–*	*–*	*–*	*–*	*–*	*–*	*–*	*–*	*–*	*–*	*–*	*–*	*–*	*–*	*–*	*–*	*–*	*–*
20:5n-3 (EPA)	*–*	*–*	*–*	*0.1*	*–*	*–*	*–*	*–*	*–*	*–*	*–*	*–*	*–*	*–*	*–*	*–*	*–*	*–*	*–*	*–*	*–*	*–*	*–*
22:5n-3 (DPA)	*–*	*–*	*–*	*0.1*	*–*	*–*	*–*	*–*	*–*	*–*	*–*	*–*	*–*	*–*	*–*	*–*	*–*	*–*	*–*	*–*	*–*	*–*	*–*
22:6 n-3 (DHA)	*–*	*–*	*–*	*0.2*	*–*	*–*	*–*	*–*	*–*	*–*	*–*	*–*	*–*	*–*	*–*	*–*	*–*	*–*	*–*	*–*	*–*	*–*	*–*
***Total n-3 PUFA***^***4***^	***0.8***	***2.7***	***1.7***	***1.5***	***0.9***	***0.2***	***0.8***	***0.3***	***36.6***	***–***	***0.9***	***37.6***	***0.5***	***0.1***	***57.1***	***0.8***	***0.2***	***9.1***	***1.2***	***0.5***	***7.7***	***0.1***	***13.2***
***Total PUFA***^***5***^	***2.3***	***18.9***	***34.0***	***11.3***	***14.5***	***23.5***	***12.2***	***59.5***	***56.7***	***2.2***	***55.0***	***74.4***	***69.1***	***21.8***	***69.9***	***12.5***	***12.3***	***28.4***	***33.8***	***44.5***	***62.0***	***61.2***	***73.2***
***n-3/n-6***	***0.53***	***0.17***	***0.05***	***0.15***	***0.07***	***0.01***	***0.07***	***0.01***	***1.82***	***0.00***	***0.02***	***1.02***	***0.01***	** < *0.01***	***4.43***	***0.07***	***0.02***	***0.47***	***0.04***	***0.01***	***0.14***	***0.00***	***0.22***

^1^includes iso15:0, anteiso15:0, 15:0, iso16:0, iso17:0, anteiso17:0, 17:0, iso18:0, 19:0

^2^includes 14:1, 16:1n-9, 17:1, 20:1n-7.

^3^includes 20:3n-6, 22:4n-6.

^4^includes 20:2n-3, 21:5n-3.

^5^includes 16:2, 16:3, 16:4

– not detected.

OA, oleic acid; LA, linoleic acid; ARA, arachidonic acid; ALA, alpha-linolenic acid; EPA, eicosapentaenoic acid; DPA, docosapentaenoic acid; DHA, docosahexaenoic acid.

Fatty acids have been previously used to distinguish between wild and cultured fish^58^. In a study examining the fatty acid profiles of farmed salmon over a 10-year period the authors charted an increase in OA, LA and ALA, which they associated with an increase in vegetable oil use, primarily rapeseed, over the same period^37^. These same fatty acids, especially LA, were higher in farmed fed seafood than wild (Supplementary Figs. 1–3), with the type of oil used influencing the levels of these vegetable ‘marker’ fatty acids. Seafood sticks (surimi), the only processed seafood product analysed in this study, also exhibited higher levels of these fatty acids owing to the inclusion of vegetable oil, namely rapeseed, as a main ingredient alongside whitefish such as Alaskan pollock (*Theragra chalcogramma*) and hake (*Merluccius spp*.). Nevertheless, the use of marker fatty acids should be interpreted with caution when examining the provenance of food. For example, the high level of OA in swordfish (*Xiaphius glophius*) may indicate a farmed fish fed vegetable oil, but swordfish are not farmed and OA levels are known to vary naturally throughout different anatomical regions of swordfish^59^. Wild flathead grey mullet (*Mugil cephalus*), on the other hand, presented both a LA level and *n*-6/*n*-3 ratio comparable to farmed fish. Although grey mullet is also farmed, there are instances where the fatty acid profiles of wild mullet can be altered through consumption of waste feed and faeces from their close aggregation to fish farm cages or from other anthropogenic sources such as sewage outlets, to be similar to farmed fish^60,61^. One further cautionary point is that, even if the general trend is for increased inclusion of vegetable oils in feeds for farmed fish, formulations can vary greatly based on species requirements, but also within the same species according to individual producer specifications that can result in some fish feeds being high in fish oil while others contain more vegetable-derived oils^38^.

**Fig. 3:**
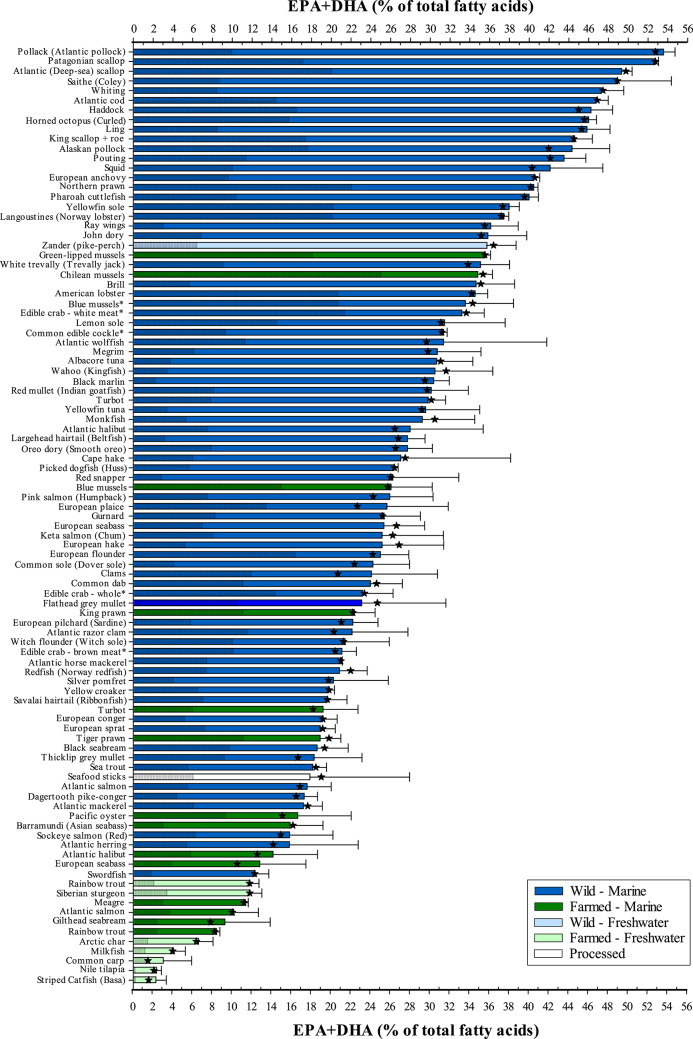
Proportion of EPA and DHA (% of total fatty acids) of wild and/or farmed seafood of marine and/or freshwater origin. Samples ranked in descending order. ★ indicates median EPA + DHA value. Shaded portion of bars denotes contribution of EPA to total EPA + DHA level (% of total fatty acids). All samples analysed raw unless denoted by *. Refer to Supplementary Table 1 for further sample information.

The way in which oil sources can be used are largely dependent upon the species farmed. For many freshwater species such as carps or tilapias EFA requirements can be generally satisfied, as in humans, through the supply of ALA and/or LA, whereas marine species tend to have a requirement for the LC-PUFA, EPA, DHA and DHA^41^. Primary production of EPA and DHA in nature occurs within marine microalgae and other single cell microbes, and they are then transferred and magnified through the food chain^62^. Unlike their wild counterparts in the marine environment, where there is an abundance of *n*-3 LC-PUFA, farmed marine fish rely upon the inclusion of these fatty acids in the feed provided. However, oilseed plants lack both EPA and DHA (see Table 2), and their use within aquaculture has resulted in a diminishing level of both these fatty acids in the feeds and, consequently, flesh of farmed fish^35–38^. Levels of EPA + DHA, as a proportion of total lipid were, therefore, predictably lower in farmed species of fish, irrespective of marine or freshwater origin, reaching a low of 2.3 ± 1.1% in the freshwater-reared striped catfish (*Pangasianodon hypophthalmus*), also known as the Vietnamese river cobbler or basa, and 8.4 ± 0.3% for marine-reared rainbow trout (Fig. 3). At the other end of the scale wild pollack (Atlantic pollock, *Pollachius pollachius*) presented an EPA + DHA level of 53.5 ± 1.2%. Interestingly, leaner species of wild seafood tended to have a higher proportion of EPA + DHA in their lipid. This is perhaps unsurprising as leaner animals have relatively lower lipid depots, i.e. triacylglycerol, and greater membrane phospholipid levels which, in fish tissues, are rich in EPA and DHA, which help provide an optimised environment for membrane-bound receptors and enzymes together with facilitating ion transport, endocytosis and exocytosis^63^, as well as plasmalogens that are typically higher in invertebrates, i.e. shellfish^41^. Thus, it may appear that leaner wild seafood provides more EPA and DHA than oilier and/or farmed seafood. However, consumers do not eat proportions of fatty acids, a fact often overlooked when assessing the nutritional value of food, which can lead to egregious misinformation being conveyed. Rather, it is the combination of lipid content together with lipid class and fatty acid composition that ultimately determine the amounts of fatty acids delivered to the human consumer.

### EPA and DHA delivery to consumers

**Fig. 4:**
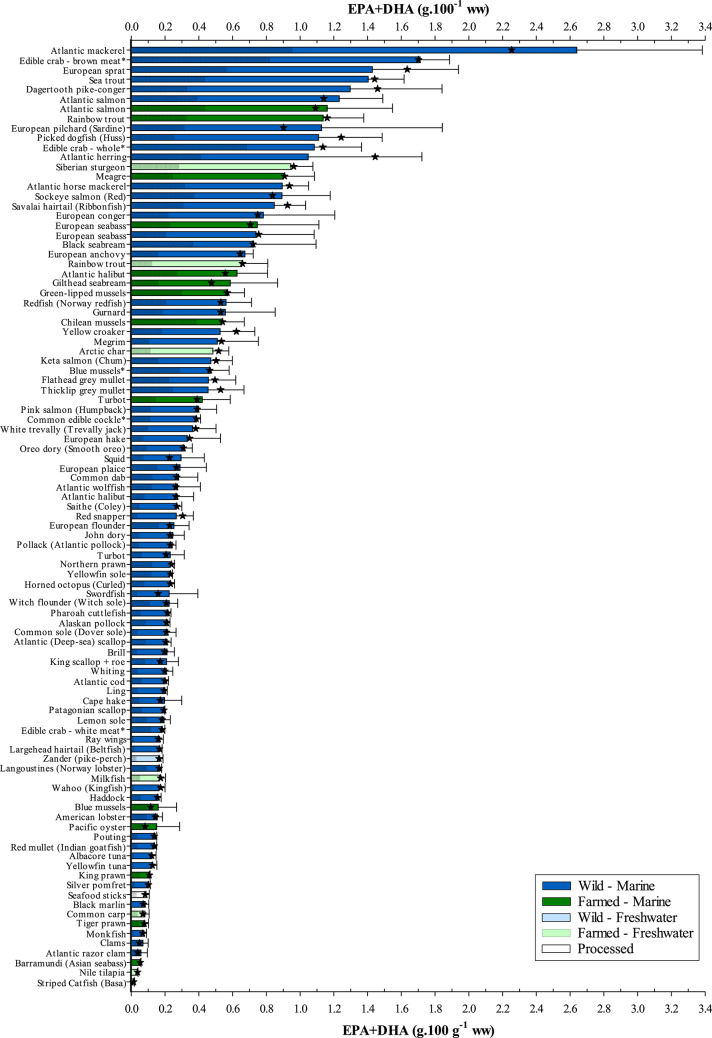
Absolute EPA + DHA contents (g.100 g^−1^ ww) of wild and/or farmed seafood of marine and/or freshwater origin. Samples ranked in descending order. ★ indicates median EPA + DHA content. Shaded portion of bars denotes contribution of EPA to total EPA + DHA contents (g.100 g^−1^ ww). All samples analysed were raw unless denoted by *. Refer to Supplementary Table 1 for further sample information.

Many indices can be used to assess the nutritional and/or health value of fatty acids in dietary fat including the index of atherogenicity and/or thrombogenicity among others^64^. The near exclusivity of EPA and DHA in marine foods and their importance to human health has resulted in the EPA + DHA index being routinely used to determine the nutritional value of seafood and related products. This differs from the fish or flesh lipid quality (FLQ) presented previously, which examines EPA + DHA levels as a percentage of total fatty acids, as it considers the more important absolute levels of EPA + DHA supplied through consumption. In the present study, wild marine Atlantic mackerel (18.1 ± 3.9% lipid) was shown to provide the most EPA + DHA, per nominal weight, of all species examined with a mean value of 2.63 ± 0.75 g.100 g^−1^ flesh wet weight (ww). In contrast, the leaner freshwater farmed striped catfish (1.1 ± 0.2% lipid) contained near-negligible levels of just 0.01 ± 0.00 g EPA + DHA.100 g^−1^ ww (Fig. 4). In fact, EPA + DHA contents generally mirrored that of lipid, albeit with some slight shifts according to environment (marine *v*. freshwater) and origin (farmed *v*. wild). While phospholipids, as components of cell membranes where EPA and DHA have important functional roles^41,65^, can also reflect dietary fatty acid composition it is to a lesser extent than storage lipid^66^. Therefore, it is the high contents of the energy storage lipid, triacylglycerol, in lipid-rich fish that dictates the absolute contents, which in marine waters is high in *n*-3 LC-PUFA due to the abundant availability of EPA and DHA throughout the food chain. Thus, selecting oily seafood species, as currently advised, would ensure that these beneficial fatty acids are provided in the diet. However, seafood is also recognised as being a rich source of protein, minerals, and other micronutrients and is highly recommended to be included in the diet by global authorities as part of a healthy balanced diet. Leaner whitefish species and shellfish (e.g. cod, haddock (*Melanogrammus aeglefinus*), ling (*Molva molva*), blue mussels (*Mytilus edulis*) and langoustines (*Nephrops norvegicus*)) for example, contain higher contents of iodine, an essential trace element of significance for human health and development, than oily fish species such that a portion each of the highest lean and oily fish species (haddock and Atlantic mackerel, respectively) would supply two-thirds of the weekly recommended intake^67^. Even so, many of these nutrients are obtainable in other foods consumed in the diet, whereas both EPA and DHA are almost exclusive to seafood.

Since the early studies linking a diet rich in marine oils to improved health outcomes^6,7^, and the subsequent discovery of the benefits of *n*-3 LC-PUFA^8–10^, advisory bodies have sought to provide the public with evidence-based recommendations. The primary focus has been coronary heart disease (CHD), founded upon early intervention and dietary-focussed studies that demonstrated decreased risk of mortality from CHD with increased fish intake^16,17^, leading to seafood being recommended within the diet^18–23^. Similarly, epidemiological and dietary intervention studies provided data that assisted policy makers to set recommended EPA + DHA intakes, although here there is less agreement with levels ranging from 0.25 to > 1 g.day^−1^^23–27^. Both EPA and DHA exert a range of biological activities, some overlapping and others distinct, such that no distinction has been made regarding intakes other than during the vulnerable periods of pregnancy, lactation and infancy where a DHA intake of up to 200 mg.day^−1^ is advised to support optimal neurodevelopment and function^9,25^. Additionally, another *n*-3 LC-PUFA, docosapentaenoic acid (DPA, 22:5*n*-3), a product of EPA elongation and precursor to DHA, is also suggested to have beneficial health effects^68^. Nevertheless, although contributing up to 7% of total lipids in flatfish species (0.06 g.100 g^−1^ ww) or 0.25 g.100 g^−1^ in the daggertooth pike conger (*Muraenesx cinerus*), DPA is not currently considered as a reliable predictor for CHD or total mortality in the same way as EPA and DHA^69^.

**Fig. 5:**
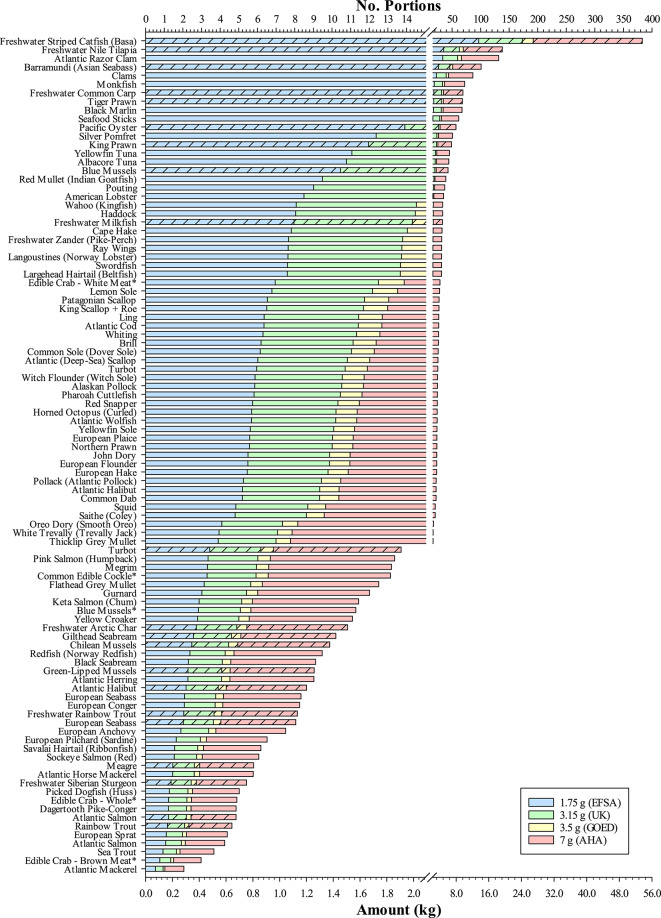
Number of 140 g portions, or amounts (g), per week required to attain the different global recommendations for EPA + DHA intake: EFSA, 1.75 g.week^−1^^25^; UK, 3.15 g.week^−1^^23^; GOED/ISSFAL, 3.50 g.week^−1^^24,27^; and AHA, 7 g.week^−1^ for those with history of cardiovascular disease^26^. Samples ranked in descending order. Species bars with shaded diagonal lines indicate farmed origin. All species are of marine origin unless stated otherwise. All samples analysed were raw unless denoted by *. Refer to Supplementary Table 1 for further sample information.

Translating the current data into a more meaningful form, based on a weekly EPA + DHA intake of either 1.75 g (EFSA^25^), 3.15 g (UK^23^), 3.5 g (ISSFAL/GOED^24,27^), and 7 g (AHA, if previously suffered CHD^26^), reveals that only Atlantic mackerel delivers the 3.15 g in a single 140 g portion as recommended by the UK government (Fig. 5). For the next highest provider, brown meat of edible crab, more than one portion (186 g), on average, would be needed. Leaner fish species, popular in the UK, such as Atlantic cod and haddock would require around 11 (1.6 kg) and 14 (2.0 kg) portions, respectively, whereas farmed Nile tilapia (*Oreochromis niloticus*) would take 61 portions, equivalent to 8.6 kg. However, farmed striped catfish (basa) demands a staggering 172 portions (24 kg) to attain this level. Regardless, it should be remembered that both tilapia and basa are lean freshwater species and are cultured to provide primarily a valuable protein source. Irrespective of species, greater amounts would obviously be needed to satisfy the higher recommended levels issued by ISSFAL/GOED and AHA. Nevertheless, there is often a tendency for researchers, as well as retailers and trade organisations, to use the lower recommendation set by EFSA when assessing the healthiness of fish products. Yet, EFSA directs individual member states to issue their own fish consumption advice, due to the varying availability of fish species and consumption behaviour among European countries^19^.

**Fig. 6:**
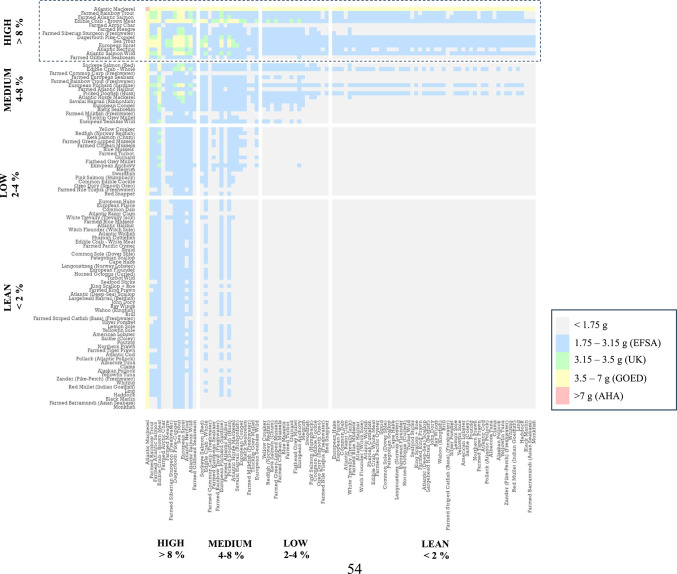
Combination of any two portions of seafood, including two of the same species, and the weekly recommended EPA + DHA intake achieved, based on average EPA + DHA contents: EFSA, 1.75 g.week^−1^^25^; UK, 3.15 g.week^−1^^23^; GOED/ISSFAL, 3.50 g.week^−1^^24,27^; and AHA, 7 g.week^−1^ for those with history of cardiovascular disease^26^. Dotted outline highlights combinations following the current guidelines of two portions of seafood per week, where at least one portion is oily. Samples ranked and grouped according to lipid content based on Ackman^39^. Portion sizes based on the UK’s 140 g recommendation^23^.

Of the 4,753 possible combinations of any two (140 g) servings of seafood from the current study, including same species, just 174 (3.7%) would provide an EPA + DHA intake equal or greater than the UK’s suggested 3.15 g.week^−1^ (Fig. 6). In fact, over 75% of the potential combinations would still deliver less EPA + DHA than the lower EFSA weekly recommended intake of 1.75 g. Atlantic mackerel, when paired with any seafood, would exceed the UK’s recommendation even surpassing the 3.50 g endorsed by ISSFAL and GOED^24,27^, while two portions of Atlantic mackerel would satisfy the 7 g.week^−1^ (1 g.day^−1^) put forward by the AHA for persons with a history of cardiovascular disease^26^. However, standard advice is for the general population to consume two portions of fish per week, of which at least one should be oily^18–23^, as these species are much richer in EPA and DHA contents. Applying this criterion to the same dataset, whereby oily species with a lipid content greater than 8% are paired with either a medium (4–8%), low (2–4%) or lean (< 2%) species, would increase the number of seafood combinations delivering above 1.75 g (EFSA) to 775 out of a possible 1,092 (71%), although only 120 (11%) and 96 (8.7%) combinations would give more than the 3.15 and 3.50 g intakes advocated by the UK and ISSFAL/GOED, respectively. Moreover, no oily species, except Atlantic mackerel, when paired with a lean seafood species would supply more than 3.15 g, while only brown meat from edible crab achieved this target when consumed with four low fat species: European anchovy (*Engraulis encrasicolus*; 3.33 g), green-lipped mussels (*Perna canaliculus*; 3.19 g), gurnard (*Chelidonichthys/Eutrigla* spp.; 3.17 g), or redfish (*Sebastes* spp.; 3.17 g). Similarly, a mere 18% of the possible 180 combinations when pairing high-fat with medium-fat species would provide more than 3.15 g. It would appear, therefore, that in order to achieve an EPA + DHA intake in excess of 3.15 g.week^−1^, at least three portions of fish should be consumed of which two must be oily. Thus, of the 7,644 combinations (three portions with two oily) available from this study, approximately 99% would exceed EFSA (> 1.75 g), 75% the UK (> 3.15 g), 61% GOED (> 3.5 g) and 1.4% > 7 g outlined by AHA (Supplementary Fig. 4). This is further supported by studies showing that increased consumption of seafood increases the omega-3 index (O3I), a measure of the percentage of EPA + DHA in red blood cells^70,71^, although amounts nor seafood species consumed were always reported. The O3I is promoted as a risk predictor of CHD, given that red blood cells reflect long-term EPA + DHA intake and that low levels are independently associated with an increased risk of CHD^72^. As such, individuals with a very low O3I < 4% are considered high risk, whereas > 8% is deemed desirable. It has been estimated that the majority of countries globally have a very low or low (> 4–6%) O3I, including the UK and USA, whereas Mediterranean countries are within the moderate range (> 6–8%) and Japan and Scandinavian countries, often renowned for being high consumers of fish, are considered to be within the desirable range^29,73^. A low dietary intake of *n*-3 should be avoided, having been attributed to a quarter of a million deaths per year in Europe alone^31^. Increasing seafood intake, therefore, is expected to have far reaching consequences beyond that of improved health outcomes, with socio-economic benefits also proposed such as saving the UK’s National Health Service (NHS) up to £600 million (approx. $768 million) per annum^32^. The question therefore is how much EPA and DHA is needed to reach a desirable level. McDonnell et al.^74^ have suggested that an average intake of 1.30 g EPA + DHA.day^−1^ (9.10 g.week^−1^) is required to achieve an O3I of > 8.0%. To put this into perspective, this is 5.2-, 2.9- and 2.6-times greater than EFSA, UK and GOED recommended EPA + DHA intake levels, respectively. If correct, then the likelihood of an individual attaining this target through seafood consumption alone is low, even when taking into the account three portions per week, of which two are oily proposed here, with two 140 g portions of Atlantic mackerel (280 g total) and one 140 g portion of European pilchard (sardine, *Sardina pilchardus*) supplying the highest EPA + DHA amount of 8.95 g, on average. Indeed, 323 g of Atlantic mackerel would need to be consumed to supply an average of 9.10 g EPA + DHA.week^−1^. Thus, it appears that further supplementation with EPA + DHA (e.g. fish oil capsules) may be essential if an individual is committed on attaining a desirable O3I. More worrying perhaps are estimations showing that the annual demand for *n*-3 LC-PUFA, based on a global population of 7 billion people consuming 0.50 g EPA + DHA.person.day^−1^ (3.50 g.week^−1^), would outstrip global annual supply by ~ 0.4 million metric tonnes^65^, equivalent to more than 2 billion people going without any EPA and DHA in their diet. It is therefore important to establish the long-term effects of seafood intake on the O3I where the type of seafood consumed and, thus EPA + DHA contents, are known to fully validate their outcomes.

While the present study highlights species we should consider including more of in our diet to increase EPA + DHA intake, there is also the wider issue of sustainability. Wild fish such as Atlantic salmon, Atlantic halibut (*Hippoglossus hippoglossus*) and European seabass (*Dicentrarchus labrax*) are rarely found, if at all, in mainstream fish suppliers and are becoming increasingly scarce to source from more specialist fishmongers. Even Atlantic mackerel, a long-time staple on the fish counter and the premier supplier of EPA + DHA from this study, is facing an increased threat with the Marine Conservation Society removing it from their green list of recommended species citing sustainability and overfishing concerns^75^. Terrestrial crops and animals have been domesticated and cultured for human consumption for millennia through intensive farming practices. The move towards the farming of seafood aims to relieve pressures on wild stocks while offering the availability of species not normally obtainable by typical consumers, thereby enabling the continual supply of a high nutritional product to support population growth and health.

### Wild versus farmed counterparts

To satisfy the growing demand from an ever-increasing global population, aquaculture, the farming of seafood, has developed to supply over half of the world’s seafood production to the table market^76^. However, aquaculture is not new dating back as far as 1000 BCE with the pond farming of low-value, low-trophic freshwater species such as carp and tilapia that still dominate global production statistics^77^. These systems traditionally relied upon natural pond organisms, or supplementary feeding, to provide sustenance to the fish. Nevertheless, stocking densities and hence productivity were low. Modern intensive aquaculture commenced around the early 1970’s with the production of cage-farmed Atlantic salmon in Scotland and Norway, growing into a global commodity with production currently just short of 3 million metric tonnes per annum^76^. This has been followed by other high-value marine carnivorous species such as European seabass and gilthead seabream (*Sparus aurata*), with their success underpinned largely by the development of commercial, formulated feeds designed to meet the nutritional requirements of the species farmed and provide optimal growth.

Despite the overall success of the industry, aquaculture often faces disproportionate criticism compared to terrestrial counterparts. One such critique is that the nutritional value of farmed fish is inferior relative to its wild equivalent. Although some discussion surrounding differences between wild and farmed seafood has been partly addressed above, the many types and contrasts in the seafood analysed can make it difficult to evaluate. Nevertheless, of the 97 different seafood ‘products’ sampled 21 were farmed, of which 8 had a wild counterpart for comparison. This included blue mussels which, as previously stated, are classed as non-fed aquaculture since they obtain their nutrients from the surrounding environment. Wild mussels are generally dredged from the seafloor whereas farmed are rope-grown and sometimes cultured alongside cage farms as part of an integrated multi-trophic set-up aimed at bioremediation, where the uptake of lipids from farm wastes can alter nutritional profiles^78^. However, a further complication in the present study is that farmed mussels were purchased raw whereas wild mussels were only available pre-cooked. Despite all efforts to obtain raw-only products many shellfish products analysed were sold as pre-cooked. Consequently, the effects of cooking may explain the differences between EPA + DHA contents of the farmed (raw) and wild (cooked) mussels, 0.16 ± 0.11 and 0.47 ± 0.11 g EPA + DHA.100 g^−1^, respectively (Fig. 4), as the loss of moisture due to cooking can increase lipid contents on a per weight basis^79^. The issue of lipid contents in wild and farmed fish has, however, been a more contentious issue with anecdotal claims that farmed are much fattier than their wild equivalents. This statement appears generally true for the current survey (see Fig. 1). High-fat (high-energy) diets are often used within intensive fish-farming to optimise production, supplying high density energy for maintenance and growth, sparing the more expensive dietary protein for incorporation into muscle protein, with any excess lipid deposited within storage organs^41^. Wild Atlantic salmon also accumulate similar lipid levels to optimised farmed salmon when caught in feeding grounds in the open ocean^80^. However, wild salmon are caught during their homeward migration when they have already utilised much of their lipid stores to supply energy for the journey and the development of reproductive organs^43^. Thus, the difference in lipid contents (8.6 ± 1.0 and 13.6 ± 2.8% in wild and farmed salmon, respectively, in this study) are most commonly the result of farmed salmon being harvested at peak nutritional condition whereas wild salmon are caught after the energy-sapping spawning migration. A similar situation may arise for wild sea trout, the native anadromous (sea-going) form of brown trout, which presented a lower lipid content (9.2 ± 0.6%) than marine-reared rainbow trout (16.6 ± 3.2%), but higher than freshwater-farmed trout (6.9 ± 2.2%), which also reflected differences in final harvest sizes (4 kg *v*. 400 g, marine and freshwater, respectively). For non-salmonid species, particularly Atlantic halibut (1.7 ± 1.4 and 6.7 ± 2.6%), European seabass (4.0 ± 1.4 and 7.2 ± 2.2%), and turbot (*Psetta maxima*; 1.2 ± 0.4 and 3.0 ± 1.3%, wild and farmed, respectively) the use of high-energy diets appears to result in more lipid being stored within muscle tissue in farmed individuals than in their wild forms^81,82^.

**Fig. 7:**
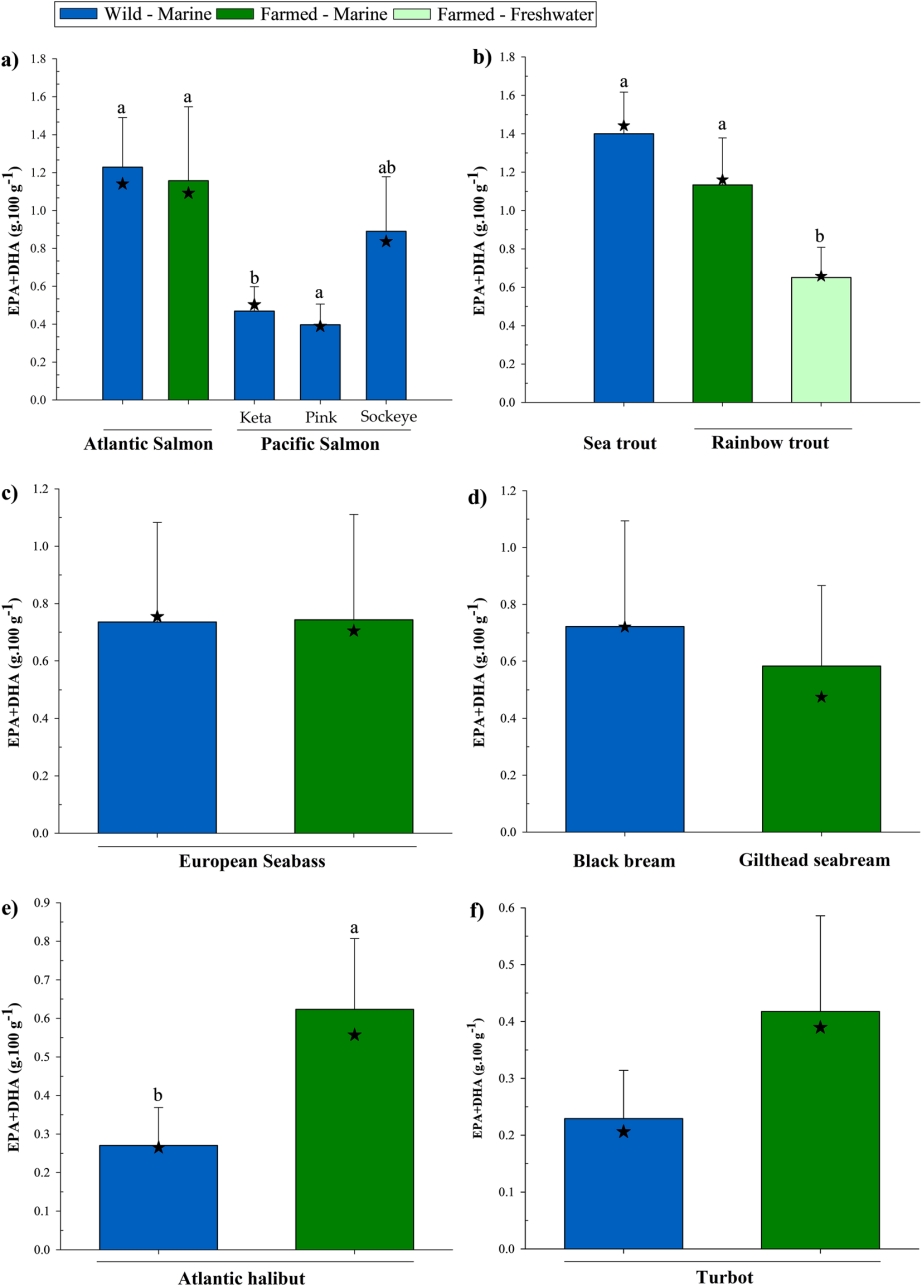
Comparison of EPA + DHA contents (g.100 g^−1^ flesh ww, mean ± SD) between farmed fish and their respective wild counterparts; (**a**) Atlantic and Pacific species of salmon, (**b**) rainbow and sea trout, (**c**) European seabass, (**d**) gilthead and black seabream, (**e**) Atlantic halibut, (**f**) turbot. Bars bearing different lettering within same species graphs indicate a significant difference (*P* < 0.05). ★ indicates the median EPA + DHA content. Note that scaling for EPA + DHA contents varies between graphs.

Diet composition is arguably the most important factor accounting for differences in flesh fatty acid profiles between farmed fish and their wild counterparts. Traditionally, farmed fish, particularly carnivorous marine species, were fed diets consisting largely of fishmeal and fish oil as protein and lipid sources, respectively, effectively mimicking the natural diet. However, this has presented the aquaculture industry with a major paradox since these ingredients are reliant upon wild capture fisheries, which are themselves stagnant or in decline. Thus, as aquaculture has grown into being a major source of EPA and DHA for humans it has also, simultaneously, become the main consumer of these fatty acids^83^. Consequently, there has been a major initiative over the past 20 + years to find alternatives to these limited and finite marine ingredients. This has focussed predominantly on the inclusion of terrestrial oils in aquafeeds which, as discussed previously, are devoid of EPA and DHA resulting in the decline of these fatty acids in the feeds and flesh of farmed seafood^35–38^. Nevertheless, EPA + DHA contents of the main farmed species are comparable, if not better, than their wild counterparts (Fig. 7a–f). Interestingly, the similar EPA + DHA levels presented here between wild and farmed Atlantic salmon (1.23 ± 0.26 and 1.16 ± 0.39 g.100 g^−1^ ww, respectively) (Fig. 7a), suggest that farmed salmon feeds were previously higher in EPA and DHA than the natural diet of wild counterparts, given that EPA + DHA contents were around 2.80 g.100 g^−1^ ww in 2005 and have been in gradual decline since as terrestrial oil inclusion increased^37^. Pacific salmon are, however, the closest commercially available wild salmon for the standard consumer. While both Atlantic and Pacific salmon are from the same family, but different genera, Atlantic salmon is a single species whereas Pacific salmon consist of seven species of which three are routinely found in UK retailers, being keta (coho, *Oncorhynchus keta*), pink (humpback, *O. gorbuscha*), and sockeye (red, *O. nerka*). Of these, sockeye delivers the most EPA + DHA, 0.89 ± 0.29 g.100 g^−1^ ww compared to 0.47 ± 0.13 and 0.40 ± 0.11 g.100 g^−1^ ww keta and pink, respectively. Although not statistically significant, an additional 77 g on average (381 g in total) in comparison to farmed Atlantic salmon (304 g) would need to be consumed for sockeye salmon to satisfy the 3.15 g recommended EPA + DHA intake^23^. Also from the same family, the closely related trout species originating from the marine environment shared statistically comparable EPA + DHA contents but would require 229 g (wild sea trout, 1.40 ± 0.22 g.100 g^−1^ ww) and 290 g (farmed rainbow trout, 1.13 ± 0.24 g.100 g^−1^ ww) to meet the UK recommendation, respectively (Fig. 7b). However, freshwater-reared rainbow trout contained a significantly lower level of EPA + DHA at just 0.65 ± 0.16 g.100 g^−1^ ww, requiring 511 g to meet the 3.15 g recommendation. This is perhaps unsurprising since, although both marine and freshwater rainbow trout contained comparable proportions of EPA + DHA in the flesh (8.9 and 11.7%, respectively) suggesting similar fish oil inclusion levels within their feeds, the lower lipid content in the muscle of freshwater-reared trout resulted in the lower absolute levels available to human consumers. Similarly, the higher lipid content of both farmed seabass and seabream coupled with the lower level of EPA + DHA in their flesh lipid (farmed 12.7 and 9.2%, compared to wild 25.3 and 18.5%, seabass and seabream, respectively), stemming from reduced marine ingredient inclusion in feeds, helped maintain an equal EPA + DHA amount to their wild equivalents (Fig. 7c and d, respectively). In contrast, both farmed halibut and turbot contained higher EPA + DHA contents than their wild counterparts (halibut: 0.62 ± 0.18 *v*. 0.27 ± 0.10 and turbot: 0.42 ± 0.17 *v*. 0.23 ± 0.08 g.100 g^−1^ ww, farmed and wild, respectively), although the latter was not statistically significant (Fig. 7e and f, respectively). While higher lipid contents in both these farmed species would have contributed to higher absolute EPA + DHA levels, one further explanation as to why these farmed variants contained twice as much as EPA + DHA than their wild alternatives could be down to a higher level of fish oil inclusion level than found in feeds for other farmed species. Farmed halibut and turbot production is just a fraction of that for more established species such as seabass or salmon^76^, meaning that feed producers can formulate diets with higher fish oil content while using low amounts overall to feed a smaller production volume.

The inclusion of EPA and DHA in the diets of farmed fish is important, not only from a human health perspective, but also in terms of overall fish health and performance. For example, although EPA + DHA requirement of salmon is recognised commonly as around 0.5% of the feed^84^, an increasing number of studies have shown that higher levels improve the robustness of fish with suggestions that up to 3.5% of the diet is required in challenging, but essentially normal, farming conditions^85–88^. Limited supplies of fish oil have, therefore, required the aquaculture sector to seek out novel alternative EPA and DHA sources. However, EPA and DHA are derived principally from the marine environment and potential replacements such as krill or mesopelagic fish are restricted by the same sustainability issues as regular fish oil. Microalgae, along with some other single cell organisms, are the primary producers of *n*-3 LC-PUFA in the oceans where they are then accumulated and magnified through the trophic levels of the food chain^62,83^. Accordingly, much focus has centred around the cultivation and extraction of oil from lipid-rich EPA and/or DHA containing microalgae, using technology based on the heterotrophic fermentation of species used within the infant formula sector for provision of DHA. An alternative solution has been made possible through advances in gene biotechnology, whereby oilseed crops such as canola or *Camelina sativa* have been engineered through transformation with microalgal genes to produce EPA and/or DHA from the *n*-3 precursor ALA^89^. Both microalgal and GM oilseed sources show promising results when used in farmed fish^90–92^, enabling the potential to maintain and/or increase EPA + DHA contents to ensure that farmed seafood continues to supply consumers with these beneficial fatty acids.

### Consumer issues

**Fig. 8:**
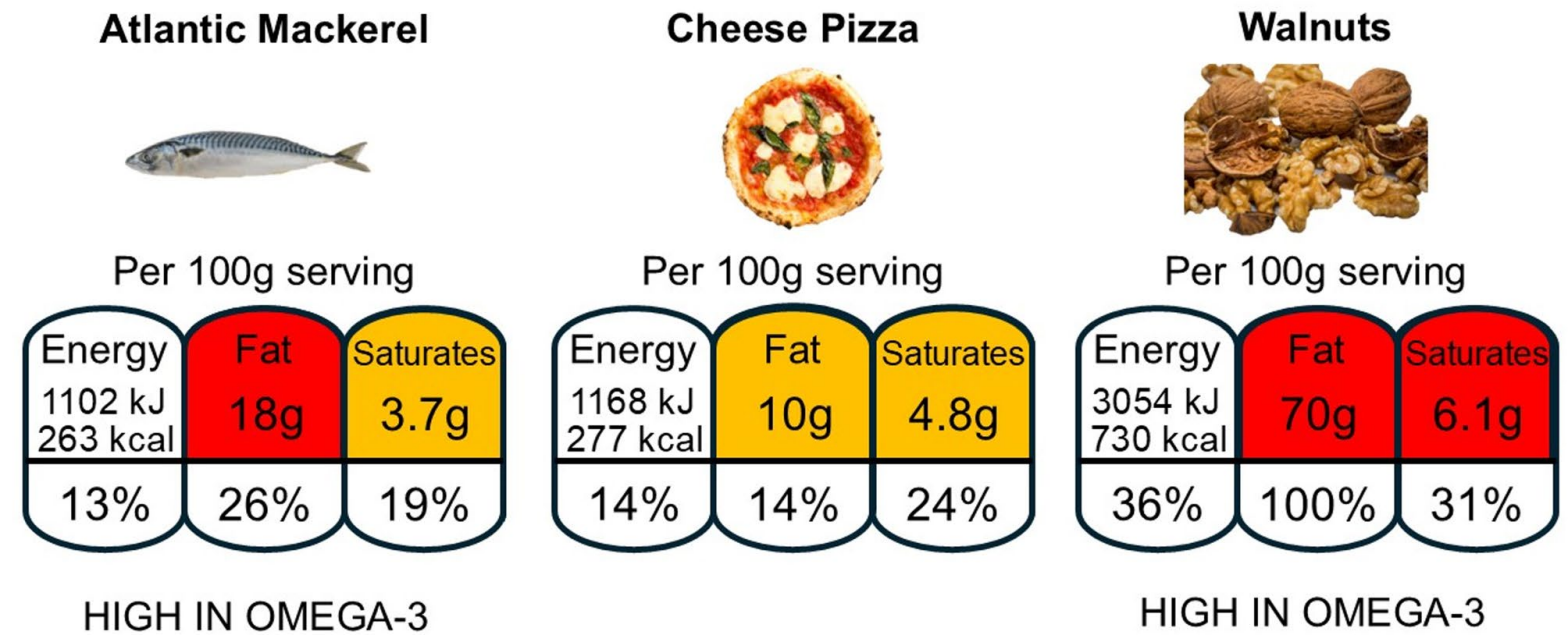
Example of front-of-packaging comparing nutritional data between Atlantic mackerel (*Scomber scombrus*), a standard cheese pizza and walnuts to illustrate potential issues consumers face when making healthy food choices. Nutritional information based on data from this study (Atlantic mackerel) and McCance and Widdowson’s *The Composition of Foods* (pizza and walnuts)^93^.

Although the benefits of including seafood in the diet are generally well engrained in the population, from the consumer viewpoint it can be difficult, as outlined above, to fully appreciate differences in the nutritional quality of fish and shellfish. Even the more experienced and ardent researcher can lack an appreciation of the factors impacting live animal compositions. Food databases such *McCance and Widdowson’s The Composition of Foods* can provide valuable nutritional information^93^. However, maintaining and updating food datasets is important to ensure that data remain relevant while embracing changes to eating habits and other nutritional developments^94^. For example, it has been over 10 years since the UK’s fish and fish products dataset was last updated^95^. In the interim period, a wider selection of seafood, of both wild and farmed origin, has become available to consumers, together with changes in farming practices altering the nutritional composition of cultured seafood, particularly with respect to EPA + DHA contents^35–38^. Similarly, climate change is predicted to negatively affect EPA and DHA production in phytoplankton and, therefore, availability throughout the wider food chain^34^. Moreover, the present study has also provided information on where species were caught or farmed, since nutrient availability can differ between wild fishing ground locations, and farmed dietary formulations may vary between production regions, both of which can affect greatly the nutritional composition of individuals of the same species^38,67,96^. Consumers are, nevertheless, able to consult the back of retail packaging for nutritional information although, in terms of *n*-3 content, this can still be confusing since total *n*-3 may be reported, which includes the sum of ALA and EPA + DHA contents instead of reporting separately^38^. Front-of-package (FOP) labelling can be a quick guide for consumers not wishing or willing to spend time trawling through nutritional tables. Several countries have adopted a traffic light system allowing consumers to make more informed choices and healthier lifestyle purchases based on fat, saturated fat, sugar and salt contents whereby red refers to foods that should be eaten less often or in small amounts; amber, an ok choice; or green, a healthy choice. Here again, consumers can be misled. Atlantic mackerel from this study would be graded as red and amber for fat and saturated fat, respectively, which, at first glance, would suggest it is less healthy than a standard cheese-based pizza bearing amber for both fat and saturated fat content (Fig. 8). Conversely, FOP labelling may also highlight the nutritional or health benefit of foods, such as *n*-3 contents, whereby pre-packaged products containing ≥ 0.3 or 0.04 g.100 g^−1^ ALA or EPA + DHA, respectively, can claim to be a ‘*Source of omega-3*’ and those ≥ 0.6 or 0.08 g.100 g^−1^ ALA or EPA + DHA, respectively, as ‘*High in omega-3*’^97^. Nearly all samples surveyed in the present study could claim to be ‘*High in omega-3*’, whereas Atlantic razor clam (*Ensis directus*), barramundi (Asian seabass, *Lates calcarifer*), black marlin (*Makaira indica*), clams (*Tawera gayi*), common carp, monkfish and Nile tilapia would be labelled as the lower ‘*Source of omega-3*’. Only the striped catfish (basa) would fail to make any claim since both ALA (0.003 ± 0.001 g.100 g^−1^) and EPA + DHA (0.01 ± 0.00 g.100 g^−1^) contents fall below the threshold. While such labelling may be reassuring for some consumers, it could also prove confusing as it fails to distinguish between products rich in ALA such as walnuts which, while providing some beneficial effects, do not confer the same health benefits as products high in EPA and/or DHA^98^. Such ambiguity could potentially lead to consumers opting for more non-seafood products (ALA-rich) in the belief that they are consuming the same *‘omega-3*’ as in seafood.

**Fig. 9:**
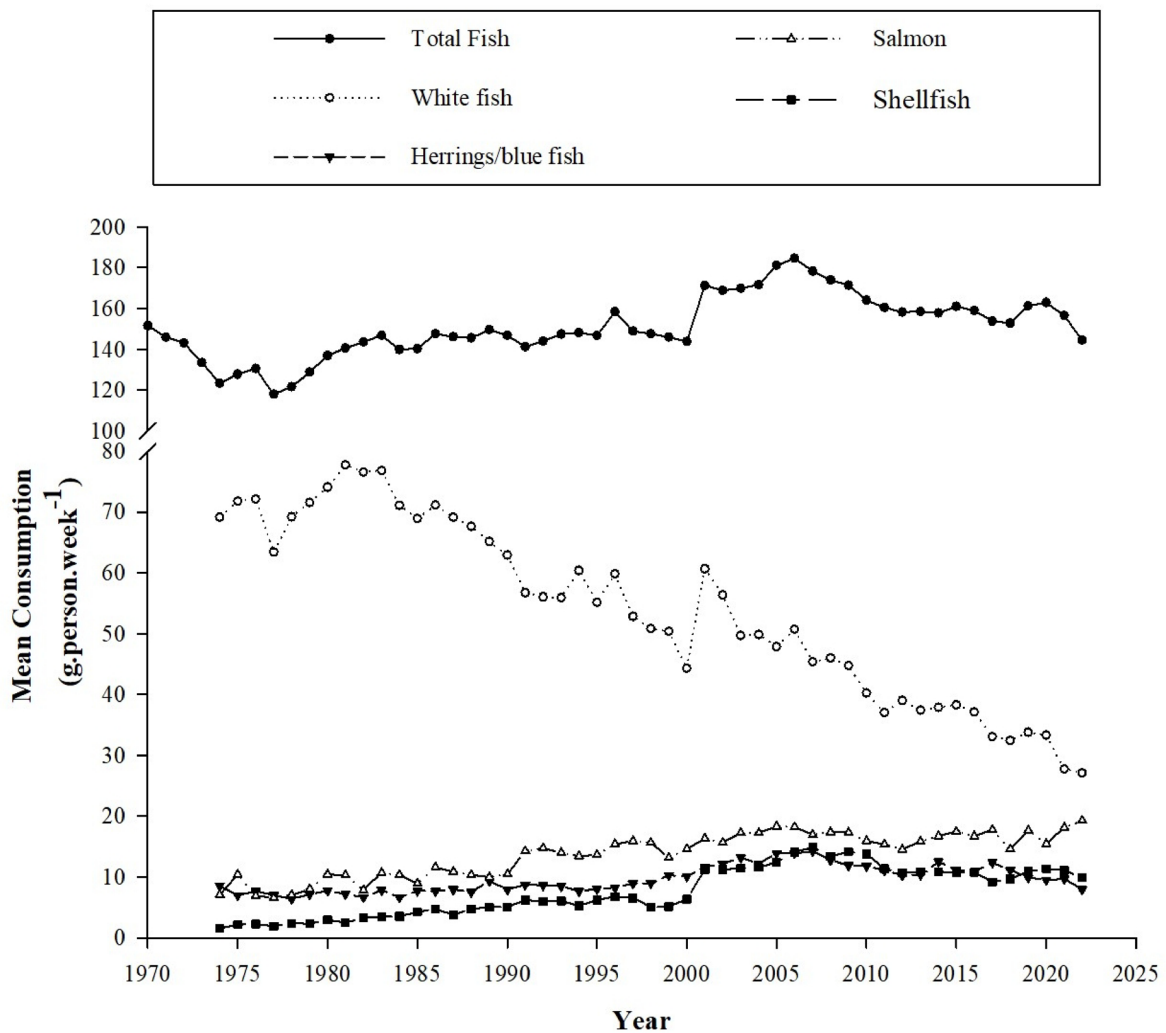
Mean UK seafood consumption (g. person^−1^.week^−1^) between 1970 and 2022. Data extrapolated from the UK’s Family food datasets for household purchases (updated 09/2023). Data from 2001 onwards also include eating out purchases (*Source*: Family food statistics, Department for the Environment, Food and Rural Affairs (DEFRA)^100^).

Despite dietary advice advocating the consumption of fish twice per week it is thought that just 20% of the global population adhere to these guidelines^99^. In the UK, national statistics shows that current average seafood consumption is just over half of the recommended level at 145 g.person.week^−1^^100^ (Fig. 9), based on an expected intake of 280 g.week^−1^ comprising typically of two 140 g servings^23^. There are several reasons why an individual may not consume enough, if any, seafood including dietary and/or lifestyle choices (i.e. vegan or vegetarianism), with costs being a commonly cited barrier together with personal preferences due to a dislike of preparing, cooking, smell and/or taste^101^. Additionally, smaller portion sizes for shellfish such as mussels are typically served per meal than that for fish^70^, or weights of individual fish fillets sold in retailers are commonly less than the recommended 140 g portion size^38^. Unlike processed foods, where final product weights can be reliably controlled, the fluid nature of animal growth, particularly for wild-caught seafood, makes this more difficult. Then there are the guidelines themselves, which may discourage certain cohorts. For instance, women who are pregnant or are of child-bearing age are told to limit their consumption of fish, particularly long-lived, higher trophic species such as swordfish and tuna, due to the risk that contaminant accumulation could pose to offspring^23^. Methylmercury, the neurotoxic organic form of mercury, accounts for around 50% of total mercury levels in lower trophic seafood, such as carps and mussels, but can be greater than 80% in higher trophic species^102^. However, recent research suggests that the net benefits gained by children from maternal fish consumption outweighs the associated risks from methylmercury contents^103^. Moreover, the overall benefits gained from seafood consumption are widely thought to outweigh any risks^23,104^. For vegans and vegetarians, those with seafood allergies and others that avoid seafood for a variety of reasons (e.g. dislike of taste, smell, bones and perception of high prices etc.), obtaining preformed EPA and DHA in the diet has always been a challenge due to their marine origin. Nevertheless, the increasing, albeit limited, availability of *n*-3 LC-PUFA from novel alternative sources such as microalgae, either in the form of supplements or food fortification, could provide much needed access to a sustainably produced alternative supply of EPA and/or DHA that was not available previously^62,83^. Furthermore, the controlled culture conditions for microalgae also have the added benefit that they are generally contaminant free^105^. Additionally, GM oilseed crops may also provide increased access to EPA and/or DHA sources, having already demonstrated comparable effects to conventional fish oil supplementation^106^. Just as with fish oil capsules, these may not necessarily supply the complete suite of beneficial nutrients obtained through fish consumption yet may be obtained through other dietary sources. However, all alternative sources face challenges including increasing production volumes, navigating regulatory concerns, and potentially high prices^62^.

## Conclusion

Seafood remains the primary dietary source of the health-promoting *n*-3 LC-PUFA, yet several challenges must be addressed to fully realise its benefits. Central to this is the need for enhanced education to raise awareness and increase intake levels. This study highlights the variability in lipid and *n*-3 LC-PUFA levels across commercially available wild and farmed species, providing valuable insights into their respective contributions to EPA + DHA intakes. Although wild Atlantic mackerel provided the highest EPA + DHA content per portion of analysed species, concerns about the sustainability of wild capture fisheries persist. Conversely, while farmed seafood faces its own nutritional challenges, including changes to farming practices that have resulted in increased *n*-6/*n*-3 ratios in farmed compared to wild and non-fed aquaculture species, it still supplies a comparable or higher amount of EPA + DHA than their wild equivalents. The overall lack of a global consensus on optimal EPA + DHA intake levels perhaps highlights a pressing need to harmonise recommendations. Nevertheless, it is clear that the current dietary recommendation advocating the intake of two portions of seafood per week, of which one should be oily, is inadequate at delivering even the lowest level of 0.25 g.EPA + DHA.day^−1^ (1.75 g.week^−1^) established by EFSA. Instead, alternative EPA and DHA sources would need to be found or revisions applied encouraging consumption of three portions of seafood in the diet, with at least two being oily fish (> 8% lipid), if consumers want to meet this lower intake level and achieve the higher albeit challenging targets.

## Materials and methods

### Sample collection and preparation

Replicate samples of ninety-seven different seafoods comprising fresh and/or frozen fish and shellfish (crustaceans and molluscs) of wild and/or farmed origin were purchased from UK retailers (fishmongers, supermarkets, online) between January 2016 and December 2022 (refer to Supplementary Table 1 for details). In addition, one processed sample of seafood sticks (surimi) was purchased for analysis. Samples of the same species (minimum 3) were obtained at different times as well as from different retailers, where available, to mitigate the risk of sampling from the same individual fish and/or catch/harvest. Samples were thawed, where required, skinned and boned or shelled, where necessary, leaving the main edible flesh that was then homogenised to a smooth pâte using an industrial food blender mixer (Robot-Coupe Blixer® 4 V.V.; Robot-Coupe, Vincennes, Cedez, France) and storing at −20 °C until analysed. All samples were raw unless otherwise stated. Large whole fish, or cuts thereof, were generally determined on an individual basis, whereas smaller-sized fish species such as sprats (*Sprattus sprattus*) and shellfish purchased at the same time were analysed on a pooled basis. Sample identities (i.e. species, wild/farmed, location) were collected from product label information available at time of purchase. The study was granted ethical approval by the Animal Welfare and Ethical Review Body (AWERB) at the University of Stirling (AWERB/167/208/New Non ASPA). The present study did not involve any purchase of, or experimentation on, any live animal.

### Lipid content and fatty acid analysis

Total lipid was extracted from samples (~ 0.5 g) in 20 volumes of ice-cold chloroform/methanol (2:1, v/v) using an Ultra-Turrax tissue disruptor (Fisher Scientific, Loughborough, UK) based on the method of Folch et al.^107^. Non-lipid impurities were isolated by washing with 0.88% (w/v) KCl before the upper aqueous layer was removed by aspiration and the lower solvent layer containing the extracted lipid dried under oxygen-free nitrogen (OFN) and left overnight in a desiccator *in vacuo* before making up to a known concentration with chloroform/methanol (2:1, v/v) containing 0.01% (w/v) butylated hydroxytoluene (Sigma-Aldrich Chemie GmBH, Steingeim, Germany), gently flushing with OFN and storing at −20 °C.

Fatty acid compositions were determined by subjecting lipid extracts (1 mg) to acid-catalysed transmethylation at 50 °C for 16 h using 2 ml of 1% (v/v) sulphuric acid (95%, Aristar®; BDH Chemicals, Poole, UK) in methanol and 1 ml toluene^108^. The resulting fatty acid methyl esters (FAME) were extracted and purified as described previously^109^, and separated and quantified by gas liquid chromatography (GC) using a Thermo Finnigan Trace GC (Thermo Scientific, Milan, Italy) equipped with a 30 m × 0.32 mm i.d. × 0.25 µm ZB-wax column (Phenomenex, Cheshire, UK), ‘on column’ injection and flame ionisation detection. Hydrogen was used as carrier gas at constant pressure (175 kPa) and initial oven thermal gradient set at 50 °C to 150 °C at 40 °C. min^−1^, then 195 °C at 2 °C.min^−1^ 205 °C at 0.5 °C. min^−1^ to a final temperature of 230 °C at 40 °C. min^−1^. Absolute fatty acid contents per g of tissue were calculated using heptadecanoic acid (17:0) as internal standard. Individual FAME were identified by comparison to an in-house marine oil (Marinol) and known standards (Restek 20-FAME Marine Oil Standard; Thames Restek UK Ltd., Buckinghamshire, UK). Data were collected and processed using Chromcard data system for Windows (Version 2.11; Thermo Fisher Scientific Inc.). Additionally, the identities of unknown fatty acid peaks were confirmed by GC mass spectrometry (MS) using a Thermo Finnigan Trace GC Ultra™, equipped with a 30 m × 0.25 mm i.d. × 0.25 µm Restek™ FAMEWAX capillary column (Thames Restek UK Ltd.), coupled to a TRACE DSQ™ MS (Thermo Finnigan, Bremen, Germany) operating in positive electron ionisation mode (EI +). Samples (1 µl) were injected on-column with the oven temperature programmed to rise from 50 °C to 150 °C at 40 °C. min^−1^, then 180 °C at 2 °C. min^−1^, 196 °C at 0.5 °C. min^−1^ to a final temperature of 240 °C at 40 °C. min^−1^. Helium was used as carrier gas and ion source and transfer line temperatures set at 200 and 250 °C, respectively. Peaks were identified from their mass spectral profiles with assistance from in-house and National Institute of Standards and Technology (NIST) Mass Spectral libraries (Version 2.0).

### Seafood contribution to recommended intake levels

The contribution of seafood to *n*-3 LC-PUFA intake, EPA and DHA, was assessed for the various weekly recommendations of 1.75, 3.15, 3.50 and 7.00 g.EPA + DHA.week^−1^, based on the 0.25, 0.45, 0.50 and 1.00 g.day^−1^ recommended by European^25^, UK^23^, global^24,27,28^, and USA (for those with history of cardiovascular disease^26^) health advisory bodies. To remove any discrepancy due to portion sizes varying by geographical region, a 140 g suggested serving size, as recommended by the UK^23^, was applied to calculate amounts delivered.

### Statistical analyses

The mean EPA + DHA contents between farmed fish and their wild counterparts were compared using one-way analysis of variance (ANOVA) with multiple comparisons made using Tukey’s post hoc test. Data were analysed using Minitab® v.21.4.1 statistical software package (Minitab Inc., Pennsylvania, USA) and were assessed for normality by the Kolmogorov–Smirnov test and homogeneity of variances using Bartlett’s test along with the examination of residual plots. Where necessary, data were transformed using the arcsine or natural logarithm transformation. A significance of *P* < 0.05 was applied to all statistical tests performed.

## Supplementary Information

Below is the link to the electronic supplementary material.

Supplementary Material 1

## Data Availability

The datasets used and/or analysed during the current study available from the corresponding author on reasonable request.
