# Supplementary material for: Evaluating the adequacy of current dietary guidelines for seafood as a source of long-chain omega-3 fatty acids
Source: Sci Rep. 2026 Mar 20;16:15190. doi: 10.1038/s41598-026-41320-w (PMC13179344; doi:10.1038/s41598-026-41320-w)
Supplement: Supplementary file 1 — Supplementary Material 1 [file 41598_2026_41320_MOESM1_ESM.docx]

**Supplementary Table 1.** List of the seafood (common and scientific names) of wild and/or farmed origin sampled in the present study. Species name and location derived from product packaging or other product information available at time of purchase.

| **Common name** | **Scientific name^1^** | **Wild/Farmed** | **Location^2^** | ***n*** |
| --- | --- | --- | --- | --- |
| ***FRESHWATER FISH*** | | | | |
| Arctic char | *Salvelinus alpinus* | Farmed | UK | 4 |
| Common carp | *Cyprinus carpio* | Farmed | Poland | 5 |
| Milkfish | *Chanos chanos* | Farmed | Indonesia | 4 |
| Nile Tilapia | *Oreochromis niloticus* | Farmed | China | 4 |
| Rainbow trout | *Oncorhynchus mykiss* | Farmed | UK | 5 |
| Siberian sturgeon | *Acipenser baerii* | Farmed | France | 4 |
| Striped catfish (Basa) | *Pangasius hypophthalmus* | Farmed | Vietnam | 5 |
| Zander (Pike-perch) | *Sander luciopersa* | Wild | Kazakhstan | 4 |
| ***MARINE FISH*** | | | | |
| ***Anguilliformes*** |  |  |  |  |
| Daggertooth pike-conger | *Muraenesx cinerus* | Wild | FAO 27 | 4 |
| European conger | *Conger conger* | Wild | FAO 27 | 4 |
| ***Clupeiformes*** |  |  |  |  |
| Atlantic herring | *Clupea harengus* | Wild | FAO 27 IV, VII | 7 |
| European anchovy | *Engraulis encrasicolus* | Wild | FAO 37.1 | 3 |
| European pilchard (Sardine) | *Sardina pilchardus* | Wild | FAO 27 (IV, VII) | 5 |
| European sprat | *Sprattus sprattus* | Wild | FAO 27 (VII) | 6 |
| ***Gadiformes*** |  |  |  |  |
| Alaskan pollock | *Theragra chalcogramma* | Wild | FAO 61, 67 | 3 |
| Atlantic cod | *Gadus morhua* | Wild | FAO 27 (I, II, IV, V) | 6 |
| Cape hake | *Merluccius capensis* | Wild | FAO 47 | 4 |
| European hake | *Merluccius merluccius* | Wild | FAO 27 (IV, VII) | 4 |
| Haddock | *Melanogrammus aeglefinus* | Wild | FAO 27 (I, II, IV, V, VII) | 5 |
| Ling | *Molva molva* | Wild | FAO 27 | 5 |
| Pollack (Atlantic pollock) | *Pollachius pollachius* | Wild | FAO 27 (IV) | 3 |
| Pouting | *Trisopterus luscus* | Wild | FAO 27 (IV, VII) | 3 |
| Saithe (Coley) | *Pollachius virens* | Wild | FAO 27 (IV, VII) | 4 |
| Whiting | *Merlangius merlangus* | Wild | FAO 27 (IV, VII) | 5 |
| ***Lophiformes*** |  |  |  |  |
| Monkfish | *Lophius piscatorius* | Wild | FAO 27 | 4 |
| ***Mugilformes*** |  |  |  |  |
| Flathead grey mullet | *Mugil cephalus* | Wild | FAO 27 | 5 |
| Thicklip grey mullet | *Chelon labrosus* | Wild | FAO 27 | 4 |
| ***Percoideri*** |  |  |  |  |
| Atlantic horse mackerel | *Trachurus trachurus* | Wild | FAO 27 (IX) | 3 |
| Barramundi (Asian seabass) | *Lates calcarifer* | Farmed | Vietnam | 6 |
| Black seabream | *Spondyliosoma cantharus* | Wild | FAO 27 | 6 |
| European seabass | *Dicentrachus labrax* | Farmed | Greece, Turkey | 6 |
|  |  | Wild | FAO 27 | 4 |
| Gilthead seabream | *Sparus aurata* | Farmed | Greece, Turkey | 6 |
| Meagre | *Argyrosomus regius* | Farmed | Greece, Turkey | 4 |
| Red mullet (Indian goatfish) | *Parupeneus indicus, P. heptacanthus* | Wild | FAO 51, 57 | 8 |
| Red snapper | *Lutjanus malabaricus, L. sebae, Pinjalo pinjalo* | Wild | FAO 71 | 4 |
| Yellow croaker | *Larimichthys polyactis* | Wild | FAO 61 | 3 |
| ***Pleuronectiformes*** |  |  |  |  |
| Atlantic halibut | *Hippoglossus hippoglossus* | Farmed | Norway | 7 |
|  |  | Wild | FAO 27 | 6 |
| Brill | *Scophthalmus rhombus* | Wild | FAO 27 | 5 |
| Common dab | *Limanda limanda* | Wild | FAO 27 IV, VII | 4 |
| Common sole (Dover sole) | *Solea solea* | Wild | FAO 27 IV, VII | 6 |
| European flounder | *Platichys flesus* | Wild | FAO 27 | 4 |
| European plaice | *Pleuronectes platessa* | Wild | FAO 27 (IV, V, VII) | 6 |
| Lemon sole | *Microstomus kitt* | Wild | FAO 27 (IV, V, VII) | 7 |
| Megrim | *Lepidorhombus whiffiagonis* | Wild | FAO 27 (IV, VII) | 4 |
| Turbot | *Psetta maxima*  *(Scopthalmus maximus)* | Farmed | France, Portugal, Spain | 5 |
|  |  | Wild | FAO 27 | 5 |
| White trevally (Trevally jack) | *Pseudocaranx dentex* | Wild | FAO 81 | 3 |
| Witch flounder (Witch sole) | *Glyptocephalus cynoglossus* | Wild | FAO 27 | 5 |
| Yellowfin sole | *Limanda aspera* | Wild | FAO 67 | 3 |
| ***Rajiformes*** |  |  |  |  |
| Ray wings | *Leucoraja naevus, Raja montagui, R. clavata* | Wild | FAO 27 (II, IV, VI, VII) | 4 |
| ***Salmoniformes*** |  |  |  |  |
| Atlantic salmon | *Salmo salar* | Farmed | UK, Norway | 78 |
|  |  | Wild | UK, Norway | 6 |
| Keta salmon (Chum) | *Oncorhynchus keta* | Wild | FAO 67 | 5 |
| Pink salmon (Humpback) | *Oncorhynchus gorbuscha* | Wild | FAO 67 | 4 |
| Rainbow trout | *Oncorhynchus mykiss* | Farmed | UK | 5 |
| Sea trout | *Salmo trutta* | Wild | FAO 27 (IV) | 4 |
| Sockeye salmon (Red) | *Oncorhynchus nerka* | Wild | FAO 61, 67 | 6 |
| ***Scombroideri*** |  |  |  |  |
| Albacore tuna | *Thunnus alalunga* | Wild | FAO 31, 51, 57, 71, 77 | 5 |
| Atlantic mackerel | *Scomber scombrus* | Wild | FAO 27 (IV, VI, VII, VIII) | 7 |
| Black marlin | *Makaira indica* | Wild | FAO 71 | 5 |
| Largehead hairtail (Beltfish) | *Trichiurus lepturus* | Wild | FAO 71 | 3 |
| Savalai hairtail (Ribbonfish) | *Lepturacanthus savala* | Wild | FAO 51 | 3 |
| Swordfish | *Xiphias gladius* | Wild | FAO 51, 57, 71, 77 | 5 |
| Wahoo (Kingfish) | *Acanthocybium solandri* | Wild | FAO 51, 57 | 4 |
| Yellowfin tuna | *Thunnus albacares* | Wild | FAO 71, 77, 81, 87 | 6 |
| ***Scorpaeniformes*** |  |  |  |  |
| Gurnard | *Chelidonichthys lucerne, C. spinosus, Eutrigla gurnadus* | Wild | FAO 27 (IV, VII) | 8 |
| Redfish (Norway redfish) | *Sebastes* spp. | Wild | FAO 27 (V) | 4 |
| ***Squaliformes*** |  |  |  |  |
| Picked dogfish (Huss) | *Squalus acanthias* | Wild | FAO 21 | 3 |
| ***Stromateoidei*** |  |  |  |  |
| Silver pomfret | *Pampus argentus* | Wild | FAO 51 | 4 |
| ***Zoarcoidei*** |  |  |  |  |
| Atlantic wolfish | *Anarhichas lupus* | Wild | FAO 27 | 4 |
| ***Zeiformes*** |  |  |  |  |
| John dory | *Zeus faber* | Wild | FAO 27 | 7 |
| Oreo dory (Smooth oreo) | *Pseudocyttus maculatus* | Wild | FAO 81 | 5 |
| **SHELLFISH** | | | | |
| ***CRUSTACEANS*** |  |  |  |  |
| American lobster | *Homarus americanus* | Wild | FAO 21 | 4 |
| *Edible crab – brown meat | *Cancer pagrus* | Wild | FAO 27 (IV, VII) | 4 |
| *Edible crab – white | *Cancer pagrus* | Wild | FAO 27 (VII) | 3 |
| *Edible crab - whole | *Cancer pagrus* | Wild | FAO 27 (VII) | 3 |
| King prawn | *Litopenaeus vannamei* | Farmed | Ecuador, Honduras, Vietnam | 4 |
| Langoustines (Norway lobster) | *Nephrops norvegicus* | Wild | FAO 27 | 4 |
| Northern prawn | *Pandalus borealis* | Wild | FAO 21, FAO 27 (I, II) | 4 |
| Tiger prawn | *Penaeus monodon* | Farmed | Vietnam | 4 |
| ***MOLLUSCS*** |  |  |  |  |
| ***Bivalves*** |  |  |  |  |
| Atlantic scallop (Deep-Sea) | *Plactopecten magellancius* | Wild | FAO 21 | 3 |
| Atlantic razor clam | *Ensis directus* | Wild | FAO 27 (IV) | 5 |
| Blue mussels | *Mytilus edulis* | Farmed | UK | 4 |
| *Blue mussels | *Mytilus edulis* | Wild | FAO 27 | 4 |
| Chilean mussels | *Mytilus chilensis* | Farmed | Chile | 4 |
| Clams | *Tawera gayi* | Wild | FAO 87 | 3 |
| *Common edible cockle | *Cerastoderma edule* | Wild | FAO 27 IV | 4 |
| Green-lipped mussels | *Perna canaliculus* | Farmed | New Zealand | 4 |
| King scallop + roe | *Pecten maximus* | Wild | FAO 27 | 3 |
| Pacific oyster | *Crassostrea gigas* | Farmed | Ireland, UK | 3 |
| Patagonian scallop | *Zygochlamys patagonica* | Wild | FAO 41 | 3 |
| ***Cephalopods*** |  |  |  |  |
| Horned octopus (curled) | *Eledone cirrhosa* | Wild | FAO 27 (IV, VII) | 5 |
| Pharoah cuttlefish | *Sepia pharaonis* | Wild | FAO 51 | 4 |
| Squid | *Loligo vulgaris, L. forbesi* | Wild | FAO 51, 57, 71 | 4 |
| ***PROCESSED*** | | | | |
| Seafood sticks (surimi) | - | - | - | 4 |

^1^Food and Agriculture of the United Nations (FAO) nomenclature (ASFIS List of Species for Fishery Statistics Purposes. Available online: <https://www.fao.org/fishery/collection/asfis/en>).

^2^Location according to product label/information provided at time of purchase and relevant FAO fishing ground (FAO Major Fishing Areas. Available online: <https://www.fao.org/fishery/area/search/en>).

All samples raw unless indicated by *

**Supplementary Figure 1.** Proportion of 18:1*n*-9 (% of total fatty acids) of wild and/or farmed seafood of marine and/or freshwater origin. Samples ranked in descending order. indicates median lipid value. All samples analysed were raw unless denoted by *. Refer to Supplementary Table 1 for further sample information**.**

**Supplementary Figure 2.** Proportion of 18:2*n*-6 (% of total fatty acids) of wild and/or farmed seafood of marine and/or freshwater origin. Samples ranked in descending order. indicates median lipid value. All samples analysed were raw unless denoted by *. Refer to Supplementary Table 1 for further sample information**.**

**Supplementary Figure 3.** Proportion of 18:3*n*-3 (% of total fatty acids) of wild and/or farmed seafood of marine and/or freshwater origin. Samples ranked in descending order. indicates median lipid value. All samples analysed were raw unless denoted by *. Refer to Supplementary Table 1 for further sample information**.**

**Supplementary Figure 4.** Combination of any three portions of seafood, where two are oily (>8 % fat), and the weekly recommended EPA+DHA intake achieved, based on average EPA+DHA contents: EFSA 1.75 g.week^-1^ [25]; UK 3.15 g.week^-1^ [23]; GOED/ISSFAL, 3.50 g.week^-1^ [24,27]; and AHA 7 g.week^-1^ for those with history of cardiovascular disease [26]. Samples ranked and grouped according to lipid content based on Ackman [39]. Portion sizes based on the UK’s 140 g recommendation.


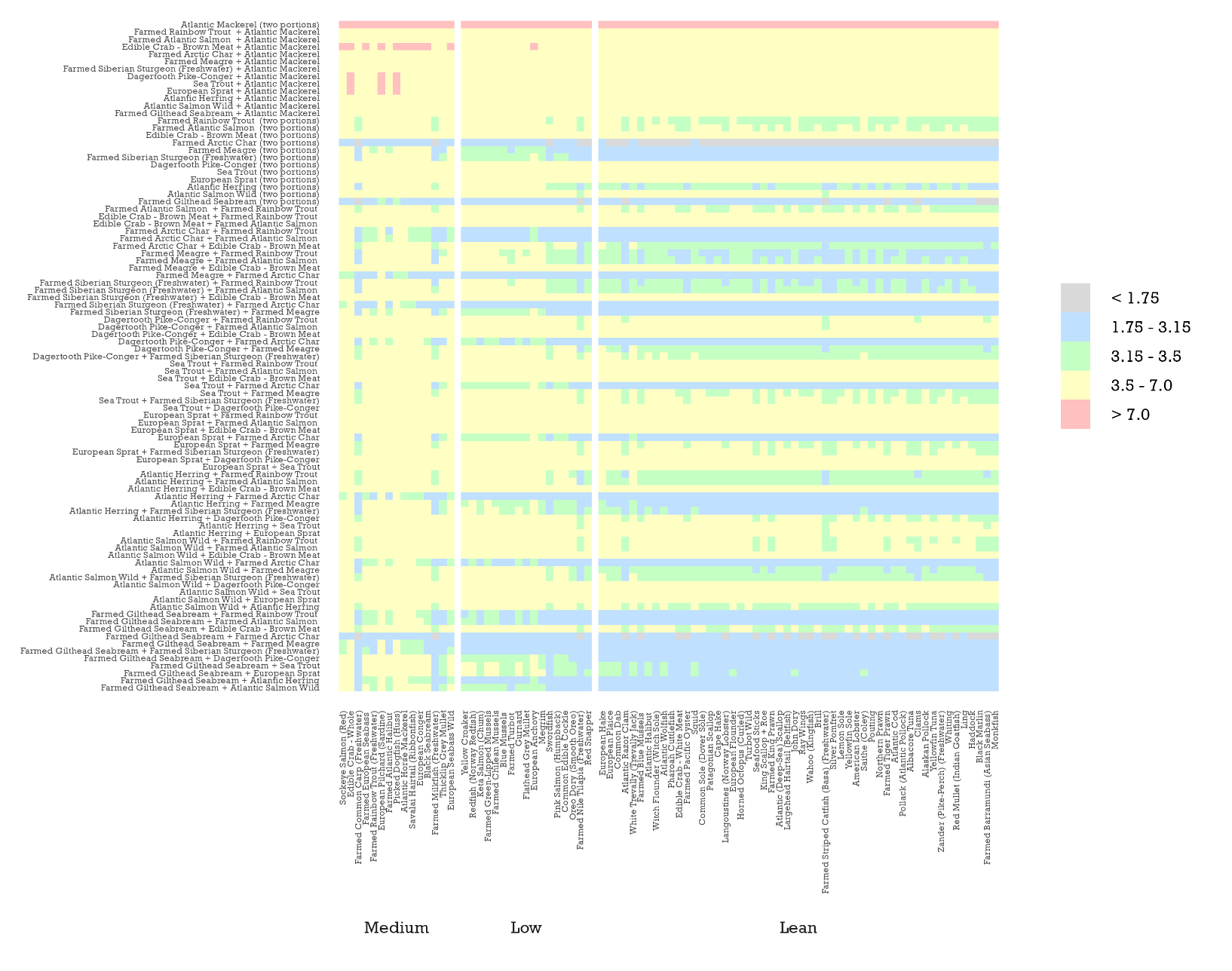

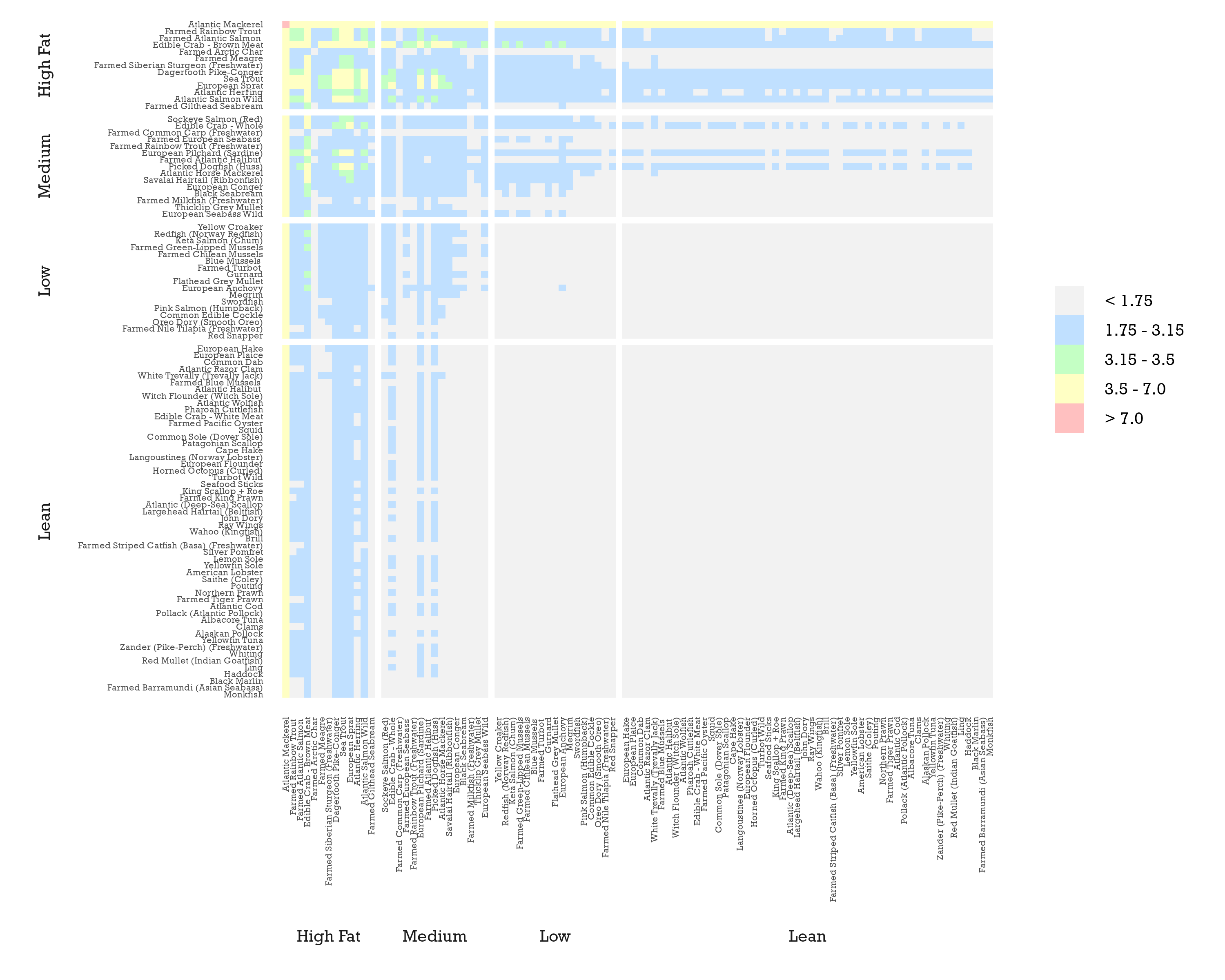


< 1.75 g

1.75 – 3.15 g (EFSA)

3.15 – 3.5 g (UK)

3.5 – 7 g (GOED)

>7 g (AHA)

**MEDIUM**

**4-8 %**

**LOW**

**2-4 %**

**LEAN**

**< 2 %**

**HIGH**

**> 8 %**
